# Drug conjugates for the treatment of lung cancer: from drug discovery to clinical practice

**DOI:** 10.1186/s40164-024-00493-8

**Published:** 2024-03-01

**Authors:** Ling Zhou, Yunlong Lu, Wei Liu, Shanglong Wang, Lingling Wang, Pengdou Zheng, Guisha Zi, Huiguo Liu, Wukun Liu, Shuang Wei

**Affiliations:** 1 Department of Respiratory and Critical Care Medicine, National Health Commission (NHC) Key Laboratory of Respiratory Disease, Tongji Hospital, Tongji Medical College, Huazhong University of Science and Technology, Wuhan, China; 2 Jiangsu Collaborative Innovation Center of Chinese Medicinal Resources Industrialization, School of Medicine, Nanjing University of Chinese Medicine, Nanjing, 210023 China; 3 Department of Geriatrics, Key Laboratory of Vascular Aging, Ministry of Education, Tongji Hospital, Tongji Medical College, Huazhong University of Science and Technology, Wuhan, 430030 China; 4 Department of Respiratory and Critical Care Medicine, Shanxi Bethune Hospital, Shanxi Academy of Medical Sciences, Tongji Shanxi Hospital, Third Hospital of Shanxi Medical University, Taiyuan, 030000 China

**Keywords:** Drug conjugates, Lung cancer, Drug discovery, Clinical practice

## Abstract

A drug conjugate consists of a cytotoxic drug bound via a linker to a targeted ligand, allowing the targeted delivery of the drug to one or more tumor sites. This approach simultaneously reduces drug toxicity and increases efficacy, with a powerful combination of efficient killing and precise targeting. Antibody‒drug conjugates (ADCs) are the best-known type of drug conjugate, combining the specificity of antibodies with the cytotoxicity of chemotherapeutic drugs to reduce adverse reactions by preferentially targeting the payload to the tumor. The structure of ADCs has also provided inspiration for the development of additional drug conjugates. In recent years, drug conjugates such as ADCs, peptide‒drug conjugates (PDCs) and radionuclide drug conjugates (RDCs) have been approved by the Food and Drug Administration (FDA). The scope and application of drug conjugates have been expanding, including combination therapy and precise drug delivery, and a variety of new conjugation technology concepts have emerged. Additionally, new conjugation technology-based drugs have been developed in industry. In addition to chemotherapy, targeted therapy and immunotherapy, drug conjugate therapy has undergone continuous development and made significant progress in treating lung cancer in recent years, offering a promising strategy for the treatment of this disease. In this review, we discuss recent advances in the use of drug conjugates for lung cancer treatment, including structure-based drug design, mechanisms of action, clinical trials, and side effects. Furthermore, challenges, potential approaches and future prospects are presented.

## Introduction

### Current status of lung cancer treatment

Cancer is a major public health problem worldwide [1–3]. Lung cancer, one of the most prevalent tumors with the highest mortality rate, originates in the trachea, bronchus and lungs, and its prevalence threatens human health [4, 5]. Systemic lung cancer therapy includes surgery, radiation and chemotherapy [6]. Surgery is used to remove some or all of the tumor tissue at the local site [7]. Radiation therapy has also been used to treat local tumors [8]. However, these approaches are ineffective in treating metastatic cancer, and the tumor cells cannot be completely removed. Many patients experience relapse after a short time following these treatments. Chemotherapy is the conventional treatment for lung cancer and provides certain benefits to patients [9]. However, chemotherapy lacks selectivity and can inhibit tumor cell growth and kill large numbers of normal cells, resulting in destruction of the immune system, many side effects and the rapid development of drug resistance [10, 11]. Chemotherapeutic drugs usually have a low therapeutic index and severe side effects, even after long-term development. Compared with traditional cytotoxic drugs, targeted therapy has higher efficacy and greater tolerability [12]. Lung cancer treatment has entered the era of precision targeted therapy. Typical molecular targeted therapy, represented by tyrosine kinase inhibitors (TKIs) of epithelial growth factor receptor (EGFR), has completely changed the treatment options for NSCLC [13]. Targeted therapy, represented by selective EGFR-TKIs, has great importance because it not only effectively inhibits tumor growth but also has fewer side effects than chemotherapy [14–16]. Although standard-of-care drugs, e.g., EGFR-TKIs, achieve a relatively high initial response in lung cancer, resistance inevitably develops after 9–12 months of treatment [17–23]. Immunotherapy, a novel treatment method that utilizes the human immune system to inhibit cancer cell growth, has received much attention in recent years. Immunotherapy does not directly interact with cancer cells but activates the immune system to eliminate tumors, thereby effectively treating lung cancer [24–29]. However, immunotherapy may easily cause side effects such as autoimmune disorders. Due to the instability of the tumor cell genome, the effectiveness of immunotherapy may vary from person to person. Therefore, developing drugs that combine the advantages of strong targeting and high toxin activity, which can simultaneously reduce toxic side effects and improve antitumor effects, has become a promising strategy for the treatment of lung cancer. Drug conjugates exhibit the above characteristics because they consist of cytotoxic drugs bound to targeted ligands via linkers, enabling targeted delivery of the drug to tumor sites. ADCs are the best-known drug conjugates and typically consist of a monoclonal antibody (mAb) bound to a payload via a linker. This construction combines the specificity of antibodies with the cytotoxicity of chemotherapeutic drugs, potentially reducing the severity of adverse reactions by preferentially targeting the payload to the tumor site [30]. For example, when ADCs enter the bloodstream, the antibody component can recognize the target and thus bind to lung tumor cells and enter through endocytosis. The cytotoxic drug is then released to kill tumor cells. In addition, the emergence and widespread use of drug conjugates may lead to the development of alternative approaches to overcoming TKI resistance [31–33].

### Developmental history of ADCs and other drug conjugates

**Fig. 1:**
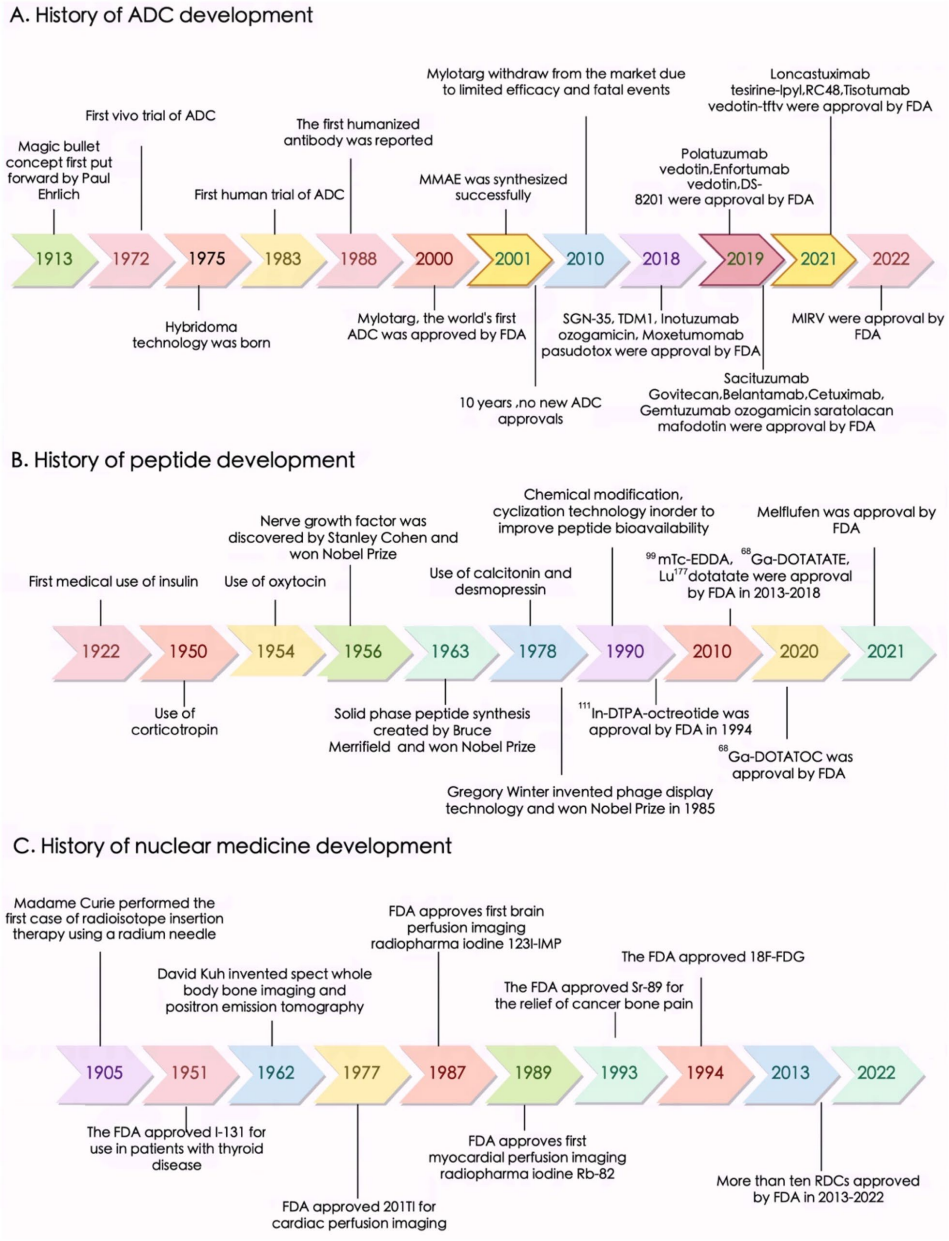
The history of drug conjugates. **A** History of ADC development. **B** History of peptide development. **C** History of nuclear medicine development. *ADC* antibody‒drug conjugate, *FDA* Food and Drug Administration, *RDC* radionuclide drug conjugate

Currently, there are many cytotoxic drugs in clinical use that can effectively kill tumor cells, but they cause numerous adverse reactions due to their lack of tumor targeting, which limits their clinical application. Therefore, instead of exploring and developing additional cytotoxic drugs, repurposing the existing nonspecific cytotoxic drugs into targeted chemotherapeutic drugs is highly important for tumor treatment. One hundred years ago, Paul Ehrlich first proposed the concept of “magic bullets”: compounds that could directly bind cancer cells [34, 35], thereby curing disease. Such compounds should be effective at killing tumors but harmless to normal cells [36]. At that time, however, research progress on antibodies was subject to technological limitations [37, 38]. In 1975, Köhler and Milstein introduced hybridoma technology, enabling the production of mAbs for therapeutic purposes [39, 40]. The research and development process present many challenges throughout [38]. For example, the molecular weight of ADCs is much greater than that of other drugs, and the ability of these drugs to penetrate the cell membrane of tumor cells is limited. A recent study showed that only a small number of the ADCs that are injected into patients can ultimately reach tumor cells. Problems with delivery, antibody specificity and antibody homology have hampered the development of ADCs. As technology continues to advance, the heterogeneity and instability of ADCs remain problematic. Moreover, mAbs that originate in mice usually have immunogenicity in the human body [41–43]. Differences between species, including differences in target structure, function, distribution, and expression levels, as well as differences in immune system function, can cause qualitative and quantitative differences in the biological responses of antibodies in experimental animals and humans. As technology advances, the selection of genetically modified animals that can express human target proteins, the use of homologous substitute antibodies, and the use of human cells or tissues for in vitro experiments have promoted the development of drugs. At present, ADCs are no longer rare, and new technologies and drug conjugate forms have emerged [44]. mAb drugs are playing an increasingly important role in cancer treatment due to their excellent targeting specificity [45, 46]. The emergence of DNA recombination technology has enabled scientists to produce engineered antibodies, leading to the development of human–mouse chimeric antibodies, humanized antibodies and fully humanized antibodies, which have overcome the problem of immunogenicity [47]. Since then, many mAbs targeting various anticancer antigens have been developed as alternatives to traditional cancer chemotherapy [48]. Currently, ADCs play a unique role in anticancer drug treatment and cannot be overlooked [49]. Gemtuzumab ozogamicin was the first ADC approved in 2000 by the FDA for the treatment of CD33-positive acute myeloid leukemia (AML) [50]. By February 2023, 12 ADCs had been approved by the FDA, 6 for hematological malignancies [51]. In addition to approved ADCs, more than 140 ADCs are currently in clinical trials for cancer treatment, reflecting and inspiring industry-wide interest in this modality [44, 52]. The successful application of ADCs has increased the enthusiasm of scientists for developing novel ADCs. However, ADCs present problems such as high molecular weight, high immunogenicity and complex antibody production processes. With the continuous progress of chemical conjugation, protein genetic engineering and other technologies, the field of targeted delivery of drug conjugates is not limited to ADCs, and various types of drug conjugates have been generated. The “formula” for the success of antibody‒drug conjugates, that is, a carrier that targets tumors, a substance that kills tumor cells and a linker that connects the first two, has provided inspiration for additional conjugated drugs. For example, a peptide‒drug conjugate (PDC) comprises a homing peptide, cytotoxin and linker [53]. In radionuclide drug conjugates (RDCs), another innovative form of medical imaging and treatment, tumor antigen-specific targeting antibodies or small molecules are connected by linkers to radioisotopes (both imaging and radiokilling), which enables accurately guided radionuclide delivery to tumors for diagnosis or treatment. Other examples include small molecule–drug conjugates (SMDCs), virus-like drug conjugates (VDCs), antibody–oligonucleotide conjugates (AOCs), antibody–cell conjugates (ACCs), immune-stimulating antibody conjugates (ISACs), antibody fragment–drug conjugates (FDCs), antibody–degrader conjugates (ADeCs), and aptamer–drug conjugates (ApDCs). In recent years, many drug conjugates have been approved by the FDA for treatment and diagnosis (Table 1). The development of ADCs and other drug conjugates from infancy to maturity over the past 100 years is depicted in Fig. 1, including advances in tumor cell-killing substances, linkers, and tumor-targeting carriers such as peptides and radioisotopes.

**Table 1:** Drug conjugates approved by the FDA

Drug name	Drug type	Mechanism	Indication	Year of FDA approval
SGN-35	ADC	SGN-35 binds specifically to CD30-positive tumor cells and releases the cytotoxic drug MMAE within the target cells [396–399]	Hodgkin lymphoma, large cell lymphoma	2011
T-DM1	ADC	T-DM1 retains the effect of trastuzumab and inhibits HER2 receptor signaling while inducing antibody-dependent cell-mediated cytotoxicity (ADCC) and inhibiting HER2 extracellular domain shedding in HER2-overexpressing human breast cancer cells [400, 401]	Breast cancer	2013
Inotuzumab ozogamicin	ADC	When the antibody binds to the CD22 receptor on the surface of B cells, the drug exerts a strong cytotoxic effect on CD22^+^ B-cell lymphoma [402–405]	Acute lymphoblastic leukemia	2017
Moxetumomab pasudotox	ADC	Internalization of moxetumumab pasudotox-tdfk leads to ribosylation of extension factor 2 ADP, inhibition of protein synthesis and apoptotic cell death [406–409]	Relapsed or refractory hairy cell leukemia	2018
Polatuzumab vedotin	ADC	Chemotherapy drugs bind specifically to the protein CD79b on the surface of B cells and release it into the B cells, thereby inhibiting cell division and inducing cell apoptosis [137, 410–412]	Diffuse large B-cell lymphoma	2019
Enfortumab vedotin	ADC	The anticancer activity of enfortumab vedotin-ejfv is due to the binding of the ADC to cells expressing Nectin-4, subsequent internalization of the ADC-Nectin-4 complex, and release of MMAE through proteolytic cleavage [270, 413–418]	Urothelial cancer	2019
T-DXd	ADC	T-DXd connects antibodies and chemotherapeutic drugs through special connectors. The antibody part can accurately locate cancer cells, deliver the chemotherapeutic drugs to the cancer cells, and accurately kill cancer cells. At the same time, it can kill adjacent tumor cells through transmembrane action [419–422]	Breast cancer	2019
Sacituzumab govitecan	ADC	Sacituzumab govitecan can deliver chemotherapeutic drugs directly to the tumor cell microenvironment by combining antibodies with TROP-2 antigen expressed on most breast cancer cells [423–427]	Triple-negative breast cancer	2020
Belantamab mafodotin	ADC	Belantamab mafodotin blmf exerts antitumor activity on multiple myeloma cells. It can kill tumor cells through MMAF-induced apoptosis, antibody-dependent cytotoxicity (ADCC) and antibody-dependent phagocytosis (ADCP) [73, 428–432]	Relapsed or refractory multiple myeloma	2020
Cetuximab saratolacan	ADC	Cetuximab saratolacan can bind to the epidermal growth factor receptor on the surface of tumor cells, prevent the receptor from binding to other ligands, inhibit the activity of tyrosine kinases, and reduce the transmission of proliferation signals to tumor cells [433]	Head and neck cancer	2020
Gemtuzumab ozogamicin	ADC	Gemtuzumab ozogamicin is a CD33 antibody drug conjugate (ADC) that, when combined with other enhanced chemotherapy regimens, can reduce disease recurrence and increase the survival of AML patients [50, 434–438]	Acute myeloid leukemia	2020
Loncastuximab tesirine-lpyl	ADC	When loncastuximab binds to CD19, the linker is degraded by proteases and releases SG3199 within tumor cells. The released SG3199 can bind to small DNA grooves, forming highly cytotoxic DNA strand cross-linking, which subsequently induces tumor cell death [436, 439–441]	Large B-cell lymphoma	2021
RC48	ADC	RC48 targets HER2 antigen on the surface of tumor cells, accurately identifying and killing tumor cells, and can cause widespread antigen release to other metastatic lesions [442–444]	HER2^+^ gastric carcinoma	2021
Tisotumab vedotin-tftv	ADC	Tisotumab vedotin-tftv is the first TF-guided ADC 1 that works by binding to TFs expressed on solid tumors [445–447]	Cervical cancer	2021
MIRV	ADC	MIRV enters tumor cells through endocytosis by binding to FRa on the surface of the tumor cell membrane, releases the anti-microtubule drug DM4 under the action of enzymes in tumor cells, and induces cell cycle arrest and apoptosis by inhibiting tubulin polymerization and microtubule aggregation [59, 70, 448–451]	Ovarian cancer	2022
^111^In-DTPA-octreotide	RDC	Octreotide is as a tumor-targeting peptide targeting the somatostatin (SST) receptor, and ^111^In is a payload that chelates with diethylene triaminopentaacetic acid [452, 453]	Diagnosis of SSTR-positive tumors	1994
^99^mTc-EDDA	RDC	^99^mTc-EDDA can be directly used for thyroid imaging or can react with a variety of chelating agents to produce different SPECT imaging agents for different nuclear medicine examination items [454–456]	Diagnostic thyroid imaging	2013
^68^Ga-DOTATATE	RDC	^68^Ga DOTATATE is a positron emission tomography (PET) radioactive tracer targeting somatostatin receptor type 2, which has been proven to be a reliable biomarker for meningioma [457–460]	Detection of neuroendocrine cancer	2016
^177^Lu DOTATATE	RDC	^177^Lu DOTATATE is a radiation therapy drug that works by binding to specific tumor-expressed somatostatin receptors. After binding to the receptor, the drug enters the cell and causes radiation damage to the tumor cells [461, 462]	Diagnosis of neuroendocrine cancer	2018
Detectnet	RDC	Detectnet is a positron emission tomography (PET) agent suitable for locating somatostatin receptor-positive neuroendocrine tumors (NETs) in adult patients	Diagnosis of neuroendocrine cancer	2020
^68^ Ga-DOTATOC	RDC	Somatostatin receptor II has become the main target for imaging and treatment of these tumors, and ^68^ Ga DOTATOC is used in diagnostic imaging for the diagnosis and treatment of NETs [463, 464]	In conjunction with PET, diagnosis of somatostatin receptor-positive neuroendocrine tumors	2020
^68^ Ga-PSMA-11	RDC	^68^ Ga-PSMA-11 PET/CT can be used to evaluate the therapeutic effect and observe the reduction in tumor lesions, especially the invasion of surrounding tissues and organs (rectum, bladder), which has great value in determining subsequent surgical indications [465–468]	Imaging for diagnosis of prostate cancer	2020
^64^Cu DOTATATE	RDC	^64^Cu DOTATATE is a radio-diagnostic agent used for positron emission tomography and computed tomography (PET/CT). It can label somatostatin receptor with nuclide to locate neuroendocrine tumors [468–471]	Locating neuroendocrine tumors	2020
Melflufen	PDC	Because of its high lipophilicity, melflufen can rapidly penetrate cancer cells, hydrolyze aminopeptidase, and effectively induce the intracellular capture of L-PAM to rapidly release alkylating agent into cancer cells [472, 473]	Multiple myeloma, ovarian cancer, breast cancer, acute myeloid leukemia, hematologic imaging	2021
Illuccix	RDC	The FDA approved Illuccix as a radiometric diagnostic agent with a ^68^ Ga- radiolabel for PSMA-PET imaging in patients with prostate cancer suspected of metastasis	Treatment of castration-resistant prostate cancer	2021
LOCAMETZ	RDC	The FDA approved Locametz as a radiometric diagnostic agent for positron emission tomography (PET) of PSMA-positive lesions [474]	Treatment of castration-resistant prostate cancer	2022
Pluvicto	RDC	The FDA approved Pluvicto for the treatment of PSMA-positive advanced prostate cancer after progression with hormone therapy or chemotherapy	Treatment of castration-resistant prostate cancer	2022

### ADCs in combination with other anticancer therapies

**Fig. 2:**
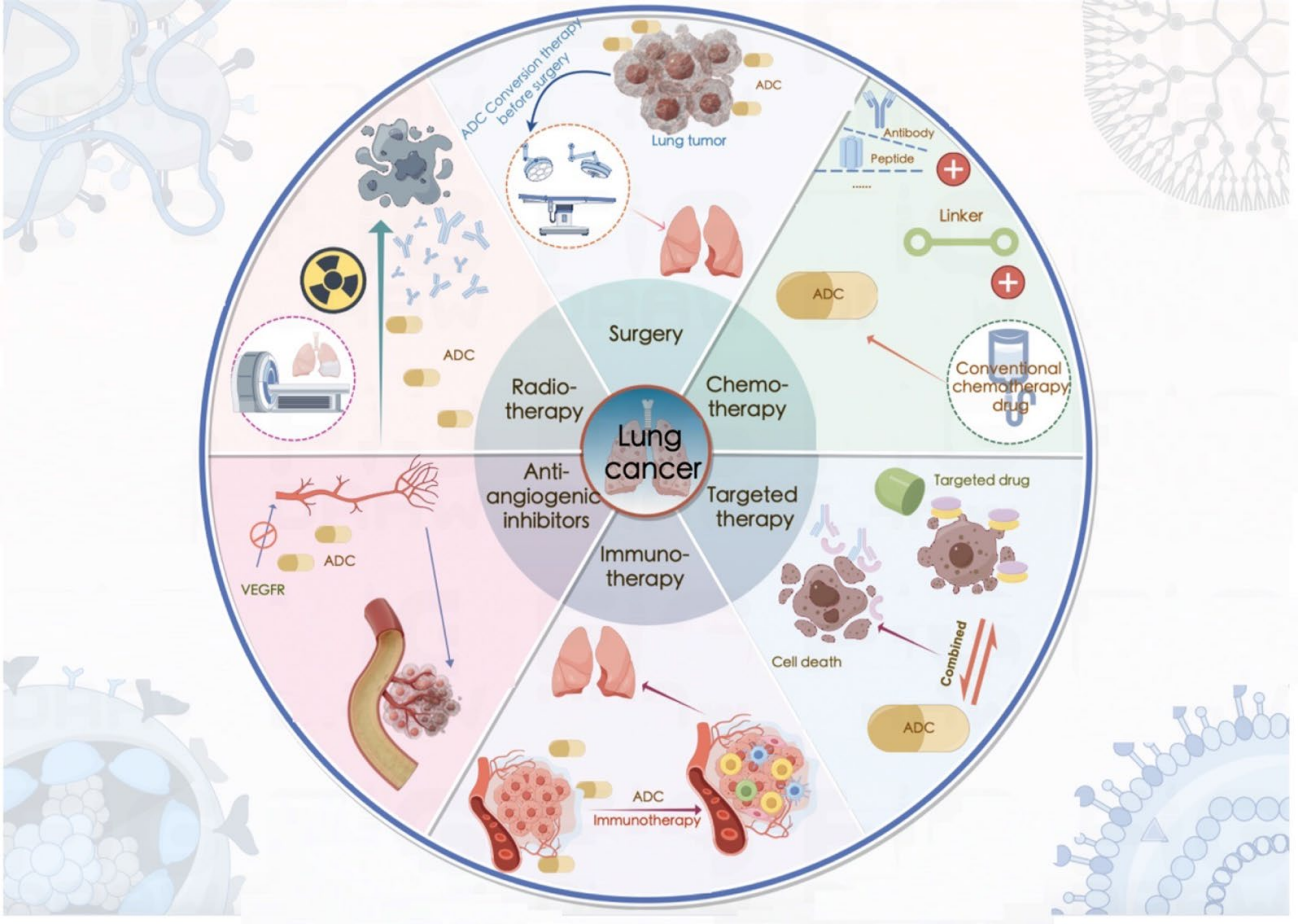
Standard-of-care treatments and combination therapy involving ADCs in lung cancer. *ADC* antibody‒drug conjugate, *VEGFR* vascular endothelial growth factor receptor

ADCs can provide survival benefits for patients. However, most patients develop resistance to ADCs and do not achieve long-lasting cancer control. Thus, ADC treatment is insufficient for many tumor types, and many ADCs are being tested in clinical trials as part of combination therapies [54]. Combining therapeutic agents may increase the likelihood of complete remission and cure [55]. The positive therapeutic effects of combination therapy have inspired the development of the next generation of ADCs in the pharmaceutical industry worldwide, and many preclinical studies and clinical trials of ADCs in combination with other anticancer drugs have been conducted [32] (Fig. 2).

Chemotherapy and ADCs act synergistically by increasing surface-antigen expression and blocking the cell cycle. Most chemotherapeutic drugs target the S phase of the cell cycle and induce G2/M arrest [56, 57]. In past trials investigating the synergistic effects of ADCs in combination with doxorubicin or carboplatin, encouraging treatment responses were observed in both platinum-sensitive and platinum-resistant ovarian cancer patients [58, 59]. The surface antigen expression of cancer cells can be affected by chemotherapy: for example, gemcitabine can upregulate HER2 expression 14.81-fold, and G2/M phase pancreatic adenocarcinoma cells are more sensitive to gemcitabine, which corresponds to a greater likelihood of gemcitabine effectively binding with trastuzumab emtansine (T-DM1) [60]. In addition, the toxicity increased when ADCs were combined with chemotherapy due to the overlap of the off-target and off-tumor effects of the payloads. Endocrine therapy is a common therapeutic approach for hormone-sensitive cancers [61]. The possibility of combining T-DXd therapy with endocrine therapy has been proven in patients with low-HER2 breast cancer at any stage [54]. Importantly, combining ADCs with endocrine therapy does not appear to increase toxicity, and endocrine therapies seem to have favorable safety profiles [61, 62]. The mechanisms of the combined application of radiotherapy and ADC therapy include the radiation-induced generation of (neo)antigens [63–65], and ADCs increase the sensitivity of tumor cells to radiotherapy, among other potential mechanisms [66, 67]. Several studies have evaluated the safety of ADCs combined with radiation, and a small number of studies have reported adverse effects (AEs) associated with radiation and ADCs [68]; however, reliable data on the effectiveness and tolerability of this combination are insufficient. Combining radiation with ADCs is a promising treatment strategy; however, additional evidence on the safety of this approach is urgently needed. The effects of combination therapy with ADCs and other targeted drugs synergistically combine multiple mechanisms, such as increased cellular uptake and antitumor activity [69–71], the upregulation of surface antigens, synthetic lethality and combined targeting, and can overcome intratumor heterogeneity and drug resistance. The evidence suggests that the efficacy of ADCs is sensitive to the efficacy of immunotherapy [72]. Numerous preclinical studies and initial findings from early-stage clinical trials have shown improved anti-tumor effects attributable to mechanisms such as Fc-mediated effector functions [73], immunogenic cell death [74, 75], epithelial cell adhesion molecule (EpCAM) [76] and the direct activation and maturation of dendritic cells (DCs) [77, 78] for combination therapy with ADCs and immune checkpoint inhibitors (ICIs) [79–81]. Research has shown that both trastuzumab deruxtecan (T-DXd) and T-DM1 can maintain the inherent efficacy of trastuzumab [82, 83]. In the KATE2 trial (phase III), AEs, including one treatment-related death, were observed in metastatic breast cancer patients treated with atezolizumab in combination with T-DM1 [84]. Reaching definitive conclusions is difficult because of the lack of a randomized design for most other studies on combining ADCs and immunotherapy, and progress in ADC combination therapy will require the identification of novel tumor targets and elucidation of their pharmacological properties (Table 2).

**Table 2:** Clinical trials of ADCs in combination with other anticancer drugs

Anticancer drugs	Target	NCT number	Drug	Partner drugs/RT type	Phase	Start	Treatment setting	Efficacy
Chemotherapy	HER2	NCT01702558	T-DM1	Capecitabine	I	2012	mBC, mGC	Negative
		NCT02073916	T-DM1	Lapatinib + Abraxane	I	2013	mBC	Positive
		NCT02073487	T-DM1	Lapatinib + Abraxane	II	2014	Neoadjuvant, BC	Positive
		NCT02562378	T-DM1	Nonpegylated Liposomal Doxorubicin	I	2015	mBC	Negative
		NCT03190967	T-DM1	TMZ	I/II	2017	mBC	Terminated
		NCT04686305	T-DXd	Durvalumab and Cisplatin	Ib	2020	mNSCLC	NA
	TROP2	NCT05687266	Datopotamab deruxtecan	Durvalumab + Carboplatin	III	2022	mNSCLC	NA
	Nectin-4	NCT03288545	Enfortumab vedotin	Pembrolizumab	I/II	2017	mUC	Positive
	TF	NCT03485209	Tisotumab Vedotin	Pembrolizumab + (Carboplatin or DDP)	II	2018	Advanced solid tumors	NA
	EGFR	NCT02573324	Depatuxizumab Mafodotin	TMZ and Radiation	III	2015	GBM	Positive
	NaPi2b	NCT04907968	Upifitamab Rilsodotin	Carboplatin	I	2021	High grade serous ovarian cancer	Terminated
	FRα	NCT02606305	Mirvetuximab Soravtansine	Bevacizumab	Ib/II	2022	High-grade epithelial ovarian	Positive
Endocrine therapy	HER2	NCT01772472	T-DM1	Unspecified	III	2013	Adjuvant, BC	Positive
		NCT04556773	T-DXd	Anastrozole or Fulvestrant	Ib	2020	mBC	NA
		NCT04553770	T-DXd	Anastrozole	II	2020	Neoadjuvant, BC	NA
	HER3	NCT05569811	Patritumab deruxtecan	Letrozole	II	2022	Neoadjuvant, BC	NA
Radiotherapy		NCT01196052	T-DM1 ± Trastuzumab	CFRT	II	2010	HER2 + early-stage BC	NA
		NCT01772472	T-DM1	CFRT	III	2013	HER2 + early BC	Positive
		NCT02573324	TMZ + Depatux-m	CFRT	III	2015	EGFR-amplification newly diagnosed GBM	Negative
		NCT02590263	TMZ + Depatux-m	CFRT	I/II	2015	EGFR-amplification grade III/IV glioma	NA
		NCT05979740	RC48 + PD-1	CFRT	II	2023	MIBRC with high HER2^+^	NA
Targeted therapy	HER2	NCT01120184	T-DM1	Pertuzumab	III	2010	mBC	Positive
		NCT03225937	T-DM1	Pertuzumab	II	2012	mCRC	Negative
		NCT02073916	T-DM1	Lapatinib + Abraxane	I	2013	mBC	Positive
		NCT01983501	T-DM1	Tucatinib	Ib	2014	mBC	Positive
		NCT02038010	T-DM1	BYL719 (alpelisib)	I	2014	mBC	Positive
		NCT02073487	T-DM1	Lapatinib + Abraxane	II	2014	Neoadjuvant, BC	Positive
		NCT02657343	T-DM1	Ribociclib	Ib/II	2016	mBC	Negative
		NCT03364348	T-DM1	Utomilumab	I	2017	mBC	NA
		NCT03523572	T-DXd	Nivolumab	I	2018	mBC & mUC	Positive
		NCT04042701	T-DXd	Pembrolizumab	I	2019	mBC & mNSCLC	NA
		NCT03975647	T-DM1	Tucatinib	III	2019	mBC	NA
		NCT04264936	RC48	Toripalimab (JS001)	Ib/II	2020	mUC	Positive
		NCT04235101	(Vic-)trastuzumab duocarmazine	Niraparib	I	2020	Advanced solid tumors	NA
		NCT04538742	T-DXd	Pertuzumab	Ib/II	2020	mBC	NA
		NCT04556773	T-DXd	Anastrozole	Ib/II	2020	mBC	NA
		NCT04539938	T-DXd	Tucatinib	II	2020	mBC	NA
		NCT04197687	T-DM1	TPIV100 + Sargramostim	II	2020	Adjuvant BC	NA
		NCT04704661	T-DXd	AZD6738	I	2021	Advanced solid tumors	NA
		NCT04983121	ARX788	Pyrotinib Maleate	II	2021	Neoadjuvant, BC	NA
		NCT04585958	T-DXd	Olaparib	I	2021	mEC	NA
		NCT05372614	T-DXd	Neratinib	I	2022	Advanced solid tumors	NA
		NCT05426486	ARX788	Pyrotinib	II/III	2022	Neoadjuvant, BC	NA
		NCT05868226	T-DXd	ALX148	I	2022	mBC	NA
	TROP2	NCT04039230	sg	Talazoparib	I/II	2019	mBC	Positive
		NCT04381832	SG	Etrumadenant + Zimberelimab	I/II	2020	mCRPC	NA
		NCT05143229	SG	Alpelisib	I	2021	mBC	NA
		NCT05006794	SG	GS9716	I	2021	Advanced solid tumors	NA
		NCT05575804	GQ1001	Pyrotinib	I/II	2022	mBC	NA
	Nectin-4	NCT04724018	EV	SG	I	2021	mUC	NA
		NCT04878029	EV	Cabozantinib	I	2021	mUC	NA
		NCT03606174	EV	Sitravatinib	II	2018	mUC	NA
		NCT04963153	EV	Erdafitinib	I	2021	Metastatic bladder cancer	NA
	FRα	NCT05200364	STRO-002	BEV	I	2022	Advanced epithelial ovarian cancer	NA
		NCT05445778	Mirvetuximab soravtansine	BEV	III	2022	Advanced epithelial ovarian cancer	NA
	MET	NCT02099058	Telisotuzumab	Osimertinib	I/Ib	2014	Advanced solid tumors	NA
	EGFR-cMET bispecific	NCT05647122	AZD9592	Osimertinib	I	2022	Advanced solid tumors	NA
	LIV-1	NCT01969643	Ladiratuzumab vedotin	Trastuzumab	I	2013	mBC	NA
	B7-H3	NCT05293496	MGC018	Lorigerlimab	I	2022	Advanced solid tumors	NA
Immunotherapy	HER2	NCT02605915	T-DM1	Atezolizumab	Ib	2015	mBC	Positive
		NCT02924883	T-DM1	Atezolizumab	II	2016	mBC	Negative
		NCT03364348	T-DM1	Utomilumab	IB	2017	mBC	NA
		NCT0303210	T-DM1	Pembrolizumab	Ib	2017	mBC	NA
		NCT03523572	T-DXd	Nivolumab	Ib	2018	mBC & mUC	Positive
		NCT04042701	T-DXd	Pembrolizumab	Ib	2019	mBC & mNSCLC	NA
		NCT05480384	T-DXd	Nivolumab	II	2022	Esophagogastric adenocarcinoma	NA
		NCT04264936	RC48	Toripalimab	Ib/II	2020	mUC	Positive
		NCT0446046	SBT6050	Pembrolizumab	I	2020	Advanced solid tumors	Positive
		NCT0511345	RC48	Sintilimab and Capecitabine	II	2021	Neoadjuvant, GC	NA
		NCT04879329	RC48	Pembrolizumab	II	2021	mUC	NA
		NCT05016973	RC48	Triplizumab	II	2021	Neoadjuvant, MIBC	NA
		NCT04873362	T-DM1	Atezolizumab	III	2021	Adjuvant, BC	NA
		NCT04740918	T-DM1	Atezolizumab	III	2021	mBC	NA
		NCT05488353	RC48	Penpulimab Injection	NA	2022	Neoadjuvant, bladder urothelial carcinoma	NA
		NCT05495724	RC48	Tislelizumab	II	2022	Bladder cancer	NA
		NCT05493683	RC48	Tislelizumab	II	2022	mCRC	NA
		NCT05333809	RC48	Pembrolizumab	II	2022	mCRC	NA
		NCT05313906	RC48	AK105 + Cisplatin	II	2022	mGC	NA
		NCT05417230	RC48	Envafolimab	II	2022	mBTC	NA
		NCT05115500	RC48	Hypofractionated RT, PD-1/PD-L1 inhibitor	II	2022	Advanced solid tumors	NA
		NCT05297552	RC48	Toripalimab	II	2022		NA
		NCT05302284	RC48	Toripalimab	III	2022	mUC	NA
		NCT05320588	BIO-106	Pembrolizumab	I/II	2022	Advanced solid tumors	NA
		NCT05514158	RC48	Chemotherapy + Nivolumab RC98	I	2022	mGC	NA
		NCT05979740	RC48	Toripalimab + RT	II	2023	MIBC	NA
	TROP2	NCT03742102	T-DXd,	Durvalumab	IB/II	2018	mBC	Positive
		NCT03337698	SG	Atezolizumab	Ib/II	2017	mNSCLC	NA
		NCT03424005	SG	Atezolizumab	Ib/II	2018	mBC	NA
		NCT03971409	SG	Avelumab	II	2019	mBC	NA
		NCT03869190	SG	Atezolizumab	Ib/II	2019	mUC	NA
		NCT04434040	SG	Atezolizumab	II	2020	Adjuvant, BC	NA
		NCT04468061	SG	Pembrolizumab	II	2020	mBC	NA
		NCT04448886	SG	Pembrolizumab	II	2020	mBC	NA
		NCT04381832	SG	Etrumadenant + Zimberelimab	I/II	2020	mCRPC	Positive
		NCT04863885	SG	IPI-NIVO	I/II	2021	mUC	Positive
		NCT05382286	SG	Pembrolizumab	III	2022	mBC	NA
		NCT05186974	SG	Pembrolizumab and a platinum agent	II	2022	mNSCLC	NA
		NCT05327530	SG	Avelumab	II	2022	mUC	NA
		NCT05687266	Dato-DXd	Durvalumab + Carboplatin	III	2022	mNSCLC	NA
		NCT05489211	Dato-Dxd	Durvalumab + AZD5305	II	2022	Advanced solid tumors	NA
		NCT04526691	Dato-Dxd	Pembrolizumab	I	2020	Advanced or metastatic NSCLC	Positive
		NCT04612751	Dato-Dxd	Durvalumab AZD2936 MEDI5752	Ib	2020	Advanced or metastatic NSCLC	NA
		NCT05941507	LCB84	Anti-PD-1	I/II	2023	Advanced solid tumors	NA
	Nectin-4	NCT03924895	EV	Pembrolizumab	III	2019	Perioperative, MIBC	NA
		NCT04223856	EV	Pembrolizumab + Cisplatin or Carboplatin	III	2020	mUC	Positive
		NCT04700124	EV	Pembrolizumab	III	2021	Perioperative, MIBC	NA
		NCT05239624	EV	Pembrolizumab	II	2022	Neoadjuvant, UC	NA
		NCT05756569	EV	Pembrolizumab	II	2023	mUC	NA
		NCT05775471	EV	Pembrolizumab	II	2023	Upper tract urothelial cancer	NA
	EGFR	NCT04305795	ASP-1929	Pembrolizumab Cemiplimab	I/II	2020	Advanced solid tumors	NA
		NCT05265013	ASP-1929	Pembrolizumab	II	2022	Locoregional recurrent SCCHNC	NA
	ROR2	NCT03504488	CAB-ROR2-ADC	PD-1 inhibitor	I/II	2018	Advanced solid tumors	NA
	FRα	NCT02606305	Elahere	Pembrolizumab	Ib/II	2022	Epithelial ovarian	NA
		NCT03835819	Elahere	Pembrolizumab	II	2019	mEC	NA
	AXL	NCT03425279	CAB-AXL-ADC	PD-1 inhibitor	I/II	2018	Advanced, refractory sarcoma	NA
		NCT04681131	CAB-AXL-ADC	PD-1 inhibitor	II	2021	mNSCLC	NA

*RT* radiotherapy, *CFRT* conventional fractionated radiotherapy, *BC* breast cancer, *TURBT* transurethral resection of bladder tumor, *GBM* glioblastoma, *MIBRC* muscle invasive bladder uroepithelial cancer, *NA* not applicable

#### Payload

The payload, also known as a “cytotoxic molecule” or “warhead” [85], is an important factor that affects the properties and activity of ADCs [86, 87]. Payload selection is crucial in the development of ADCs because the payload directly affects the therapeutic window and often plays a major role in clinical applications [88]. For success in designing therapeutic agents, cytotoxic drugs should have high cytotoxicity to tumor cells, but the amount of drug that can reach the tumor tissue after intravenous injection of an ADC is very limited, resulting in low intracellular concentrations [89]. The ideal payload should have a low molecular weight and a long half-life and should remain stable in the circulation and in lysosomes during endocytosis. Most approved cytotoxic payloads belong to one of the following three categories: microtubule inhibitors (such as maytansine or auristatin), DNA-damaging agents (such as doxorubicin, mitomycin, camptothecin analogs, and calicheamicin [90]), or topoisomerase inhibitors [91]. Cytotoxic payloads that can damage DNA are often very effective, while microtubule and topoisomerase inhibitors are moderately effective [92–95].

Microtubule inhibitors include maytansine and auristatin, both of which are derived from bacteria. Auristatin, similar to monomethyl auristatin E (MMAE) and monomethyl auristatin F (MMAF) [96, 97], is a synthetic compound extracted from the natural mitotic inhibitor dolastatin that can inhibit microtubule polymerization, leading to cell cycle arrest. MMAE can penetrate the cell membrane, and its cytotoxicity is 100–1000 times greater than that of standard chemotherapeutic drugs. In contrast, the more hydrophilic MMAF cannot penetrate the cell membrane; therefore, ADCs derived from MMAF are less efficient than those derived from MMAE, and their toxicity is relatively weak [98]. MMAE has been used in multiple ADCs. In 2015, the FDA approved brentuximab vedotin, an MMAE conjugate, for the treatment of Hodgkin’s lymphoma and anaplastic large cell lymphoma. Researchers prepared rituximab-Vc-MMAE, and the results showed high efficacy against CD20-positive cell lines but no effect on CD20-negative cell lines. In addition, rituximab-VC-MMAE was able to inhibit colony formation of CD20-positive cells. These data suggest that rituximab-c-MMAE may be an effective and selective drug for the treatment of B-cell lymphoma [99]. Bourillon et al. found that HER3 antibody‒drug conjugates (HER3 ADCs) based on MMAE were effectively internalized by tumor cells, increased the proportion of cells arrested in G2/M phase, which is the most radiation-sensitive phase in the cell cycle, and promoted programmed cell death in irradiated HER3-positive pancreatic cancer cells. HER3-ADCs reduced the clonogenic survival of irradiated cells by increasing the formation of DNA double-strand breaks (based on γH2AX levels) and regulating DNA damage repair. This approach may constitute a promising new strategy for the treatment of pancreatic cancer [100].

Maytansinoids are a class of ansamacrolides, and their derivatives are known as maytansinoidoids. Maytansinoids are natural products isolated from the African shrub *Maytenus ovatus*, and their mechanism of action is to disrupt microtubule polymerization. Maytansinoids are among the earliest cytotoxic drugs with an IC_50_ value in the picomolar range for tumor cells [101]. Maytansinoids and vinca alkaloids bind to the same sites on microtubules and have similar in vitro inhibition efficiencies. Due to their excellent stability and acceptable solubility in aqueous solutions, maytansinoids can be used to make ADCs [102, 103]. Maytansinoid derivatives are mainly divided into two types: DM1 and DM4 [104, 105]. DM1 maytansinoid derivatives (emtansine and mertansine) are potent drugs with broad lethal effects on xenografts of non-Hodgkin’s lymphoma in vivo [106]. DM4 drugs include soravtansine and ravtansine, which can enhance the “bystander effect” of adjacent cells in vivo, thereby eradicating tumors [107]. Effective payloads that act by damaging DNA include calicheamicin, doxorubicin, and camptothecin-like drugs. Unlike tubulin-binding agents, these effective payloads are not cell cycle specific and can exert cytotoxic effects on both proliferating and nonproliferating cells. Calicheamicin is a highly potent enediyne-class antitumor antibiotic originally isolated from *Micromonospora echinospora*. It can bind to the minor grooves of DNA, causing transcriptional damage, double-strand breaks, and cell apoptosis through DNA cleavage. Calicheamicin is also strongly hydrophobic, and each immunoglobulin can form drug conjugates with only a few molecules [108]. PF-06647263 is a calicheamicin-containing ADC targeting ephrin A4 and has recently entered phase I trials for triple-negative breast cancer [109]. Unlike calicheamicin, both doxorubicin and pyrrolobenzodiazepine class drugs are alkylating agents that bind to the minor groove of DNA and cause irreversible alkylation, leading to cell death [110]. A HER2-targeted ADC (trastuzumab deruxtecan, SYD985) prepared by linking trastuzumab with a new cleavable linker to the dual doxorubicin prodrug secoDUBA showed antitumor activity in preclinical breast and gastric cancer models with low HER2 expression [111, 112]. Vadastuximab talirine, a site-specific ADC currently in clinical trials, is an anti-CD33 antibody linked through engineered cysteine residues in the heavy chain that can yield a drug–to-antibody ratio (DAR) of 2. It was the first clinical ADC with a pyrrolobenzodiazepine-class drug payload and was tested in phase 1 trials in 2013 [113]. However, an efficient payload may also lead to greater safety risks. The phase III trial of vadastuximab talirine was terminated due to problems with dryness toxicity, despite achieving a complete remission rate of 70% in acute myeloid leukemia patients. The balance between efficacy and safety is a key consideration for scientists and regulatory agencies [114].

T-DXd is an ADC composed of an anti-HER2 antibody, a linker based on a cleavable tetrapeptide and a cytotoxic topoisomerase I inhibitor with cytotoxicity that has shown sustained antitumor activity in a pretreated population of HER2-positive metastatic breast cancer patients [115]. T-DXd has shown antitumor activity even in tumors with low HER2 expression. According to safety and efficacy data, the most likely recommended phase II dosage is 5.4 mg/kg or 6.4 mg/kg [116]. T-DXd also increases the antitumor immune response, as evidenced by the increased expression of DC markers, increased expression of MHC I in tumor cells and rejection of restimulated tumor cells by adaptive immune cells, indicating that T-DXDa improves the T-cell recognition of tumors. This immunostimulatory activity is distinct from the cytotoxic effect of DXd on tumor cells [117]. U3-1402 is composed of an anti-HER3 antibody (patritumab) and a DXd derivative linked together by the maleimide GGFG peptide. DX-8951 is a topoisomerase inhibitor. When U3-1402 binds to HER3 overexpressed in cancer cells, U3-1402 is cleaved by lysosomal enzymes to release DXd, which specifically inhibits topoisomerase 1 in cancer cells. Furthermore, the administration of U3-1402 significantly inhibited the growth of EGFR-TKI-resistant PC9AZDR7 xenograft tumors [118] (Table 3).

**Table 3:** Common ADC payloads

Type of toxic payload	Payload	Representative tumor-targeting ADCs
Microtubule-targeting payload	Maytansinoids	PF-06647020
	Auristatin	IMGN901, MRG003, Teliso-V, BA3011 TIVDAK, Glembatumumab vedotin, Tisotumab vedotin, EnaV
	Eribulin	BB-1701
	Tubulysins	EC1428
	Cryptophycins	Dictyostatin
	EG5 inhibitors	–
DNA-targeting payload	Enediyne	Loncastuximab tesirine-lpyl
	Topoisomerase I inhibitors	DXd
	PBD	Zynlonta
	Duocarmycins	Trastuzumab duocarmazine
RNA-targeting payload	Thailanstatin	MC-Thailanstatin A
	Amatoxins	–
Immune agonist	Toll-like receptor agonists	BDC-1001, Silverback
	STING agonists	XMT-2056
	Glucocorticoid receptor modulators	ABBV-319

*ADC* antibody drug conjugate, *PBD* pyrrolobenzodiazepines

The current cytotoxic payloads of PDCs can be divided into chemical and nonchemical agents. Chemical drug therapy is one of the three major clinical treatments for malignant tumors. Tumor chemotherapeutic drugs include alkylating agents, antimetabolites, antibiotics, hormones, plant-derived drugs, platinum-based drugs, and immunomodulatory agents. Most chemotherapeutic drugs used in PDCs for tumor treatment are alkylating agents, antibiotics, and plant-derived drugs. For example, paclitaxel can be used to synthesize PDCs, as can vincristine, doxorubicin, methotrexate, and nitrogen mustard [119]. Paclitaxel is a first-line or second-line treatment for ovarian cancer, lung cancer, and other diseases, but it can cause bone marrow suppression, cardiotoxicity and drug resistance. Conjugating paclitaxel with tumor-targeting peptides can overcome these disadvantages [120]. After conjugation with peptides, paclitaxel retains significant tumor specificity. Paclitaxel-octreotide (PTX-OCT) can specifically bind to the STTR2 receptor overexpressed in tumor tissues [121]. The main pharmacophore of vincristine is the lactone ring, and the acyl group formed after lactone ring hydrolysis can interact with the nucleophilic group of topoisomerase I. However, the lactone ring of vincristine is easily hydrolyzed and becomes inactive after entering the circulatory system. Conjugating vincristine with the integrin receptor-targeting peptide ALOS4 significantly increases the stability of its lactone ring [122]. Moreover, the ALOS4–vincristine conjugate uses a peptide moiety for targeting, thus effectively reducing the systemic toxicity of vincristine by targeted delivery to tumor tissues. Doxorubicin is a commonly used chemotherapeutic drug that induces cardiotoxicity, which is closely related to drug accumulation. Resistance to doxorubicin is very common and decreases the concentration of doxorubicin in tumor cells. Studies have shown that TAT–doxorubicin has significantly greater cell toxicity to the doxorubicin-resistant cancer cell line KB-V1 than does doxorubicin alone [123]. RGD–doxorubicin can also increase the efficiency of doxorubicin delivery to tumor cells, where it exhibits significant cytotoxicity [124].

The nonchemical agents used for cytotoxic payloads include tumor necrosis factor (TNF), small interfering RNA (siRNA), and antisense oligonucleotides (AONs). TNF is a type of cytokine secreted by macrophages or lymphocytes in the body that can kill tumor cells or cause necrosis of tumor tissues, and includes TNF-α and TNF-β. Representative PDCs targeting TNF include etanercept and infliximab. Both drugs are targeted therapeutic drugs for TNF, but the underlying mechanisms are slightly different. Etanercept is an artificially synthesized fusion protein composed of the human TNF receptor and the human IgG1 Fc region [125] that can bind to TNF and prevent its binding to TNF receptors on the cell surface, thus reducing inflammation and disease. Etanercept has been approved for the treatment of inflammatory diseases such as rheumatoid arthritis, ankylosing spondylitis, and psoriasis. Infliximab is a monoclonal antibody that can specifically bind to TNF and prevent its biological activity. Therefore, infliximab is widely used to treat clonal disease, rheumatoid arthritis, inflammatory bowel disease and psoriasis. NGR–hTNF consists of TNF-α linked to peptides containing NGR, which can specifically recognize tumor neovascularization with high CD13 expression and thus deliver high concentrations of TNF-α to exert therapeutic effects on tumor tissues. A phase II clinical trial of NGR–hTNF and doxorubicin as second-line treatments for small-cell lung cancer revealed that NGR–hTNF showed effective antitumor activity in recurrent small-cell lung cancer patients [126]. siRNA is another molecule that can be used in PDCs and has great potential in gene silencing, gene expression control, and disease treatment. A representative PDC is Atu027, which is an siRNA-targeted antibody–peptide drug synthesized using Synthesis Platform technology and is used to treat patients with liver, bile, stomach, or pancreatic cancer. In addition, siRNAs can specifically knock down specific genes, thereby interfering with their expression [127]. Researchers can design corresponding siRNAs to target genes that affect the proliferation and differentiation of tumor cells, such as epidermal growth factor receptor, caspase-3, and caspase-9 [128] (Table 4).

**Table 4:** Common PDC payloads

Type of toxic payload	Payload	Representative tumor-targeting PDCs
Chemical drug	Paclitaxel	Paclitaxel-poly(L-lysine) conjugate (PTX–PLL), ImmunoGen 853
	Amanitin	Adcetris, Kadcyla, Mirvetuximab soravtansine
	Doxorubicin	TAT-doxorubicin, RGD-doxorubicin
	MTX	Glembatumumab vedotin, NPY-MTX conjugate
	Cathinone	ALOS4-camptothecin conjugate, SG3199
	Phenylbutyric acid nitrogen mustard	Benzenebutanoic acid nitrogen mustard–AAAk conjugate, EMD 525797
Nonchemical drug	TNF	NGR–hTNF, Etanercept, Infliximab
	siRNA	cRGD–siEGFR, Atu027
	AONs	Pip-AONs, AVI-4126

*MTX* methotrexate

#### Linkers

Linkers form the chemical connection between the antibody and the cytotoxic payload in ADCs [129]. They are a critical component of ADCs, and the linker should ideally stabilize the ADC in the bloodstream, ensuring that the ADC can reach the tumor site intact and allowing cleavage and release of the cytotoxic payload when the ADC binds to the antigen or is internalized. Although the linker itself may not be cytotoxic, its stability significantly affects the toxicity of the cytotoxic molecule. Stable linkers allow the cytotoxic agent to be precisely released upon reaching the specific target, while less stable linkers are more likely to undergo nonspecific cleavage, resulting in off-target side effects. Most dose-limiting and off-target toxicity is related to the stability of the linker molecule and the release of the payload into the systemic circulation.

Two primary linker types exist: non-cleavable and cleavable [130–132]. Initially, non-cleavable linkers were thought to be more useful than cleavable linkers because they can increase the stability of ADCs in plasma [133, 134], thereby decreasing the systemic toxicity risk, expanding the therapeutic window, and increasing tolerability. However, in ADCs connected by non-cleavable disulfide linkers, such as a non-cleavable succinimidyl 4-N-maleimidomethyl cyclohexane-1-carboxylate linker connecting trastuzumab to a monoclonal antibody [135], the non-cleavable linker cannot trigger bystander effector functions, and the ADC is ineffective in tumors with heterogeneous target antigen expression [136]. Cleavable linkers are sensitive to the physiological environment. The characteristics of tumor cells or their microenvironment can be used to disassociate the payload from the antibody portion. There are several mechanisms by which these chemical linkers are cleaved: enzyme-sensitive linkers [137] include valine–citrulline (Val–Cit), glutathione-cleavable triggers, and phosphatase-cleavable triggers [138]; pH-sensitive linkers [139] include hydrazone triggers [140, 141] and carbonate triggers. In addition, there are GSH-cleavable triggers [142, 143], non-cleavable linkers, Fe(II) cleavable triggers and redox-sensitive linkers. Reducing molecules such as glutathione are usually present at higher concentrations in the cytoplasm than in the extracellular space, allowing them to cleave disulfide bonds and release the payload within the cell. ADCs containing these types of linkers also typically have better solubility than those containing dipeptide linkers. Acid-cleavable linkers are hydrolyzed by the acidic environment of endosomes and lysosomes. The recognition and hydrolysis of a protease-sensitive linker is similar to the process of a peptide sequence being hydrolyzed by lysosomal proteases [144].

Existing chemically cleavable linkers can be divided into pH-sensitive linkers, cathepsin-cleavable linkers, GSH-cleavable linkers, Fe(II)-cleavable triggers, photoresponsive cleavable linkers, novel enzyme-cleavable linkers and bioorthogonally cleavable linkers [129, 145, 146] (Table 5). Glutathione (GSH), which contains cysteine, is a small peptide present in the human body, and its concentration is significantly greater in tissues such as lung cancer tissues than in normal tissues. Glutathione-sensitive linking moieties are connected to drugs by disulfide bonds. When drugs containing such linking moieties reach tumor tissue, the linker is cleaved by glutathione, and the cytotoxic payload is released to exert antitumor bioactivity. Studies have shown that the low-pH insertion peptide-sulfur-sulfur-doxorubicin (pHLIP-SS-DOX) can target acidic tumor cells and reverse multidrug resistance [145]. Moreover, the study of in vitro cytotoxicity mediated by GSH demonstrated that pHLIP-SS-DOX has significant cytotoxicity at a pH of 6.0. Tumor cells proliferate and undergo metabolic reactions more rapidly than do normal cells, leading to lactate accumulation and a decrease in the pH in the tumor microenvironment to approximately 6.8, whereas the pH in the bloodstream is approximately 7.3. pH-sensitive linking moieties are designed to exploit this change in pH [147, 148]. Enzyme-sensitive linking moieties can remain stable in the circulatory system of the human body, but when they reach locations where they are surrounded by the target enzymes, they undergo specific enzyme cleavage. Research has shown that linking moieties containing the short peptide sequence GFLG can be specifically cleaved by tissue protease B, releasing doxorubicin in tumor cells [149]. MMPs are a family of proteases that can target the extracellular matrix, and various subtypes of MMPs are highly expressed in tumor tissues [150, 151]. MMP2 and MMP9 play important roles in tumor invasion and metastasis by degrading collagen fibers cleaved by collagenase. The short peptide sequence PLGLAG is an MMP2/MMP9-sensitive linking fragment that can be cleaved in tumor tissue [152]. Abnormal iron metabolism can elevate the levels of unbound ferrous iron [153, 154]. Spangler et al. reported the use of the Fe(II)-reactive 1,2,4-trioxolane scaffold (TRX) linker in ADCs [155]. The linker Val–Cit has been shown to exhibit widespread sensitivity to a variety of cathepsins, but only cathepsin B is thought to be highly expressed in cancer cells. Pyrophosphate groups can be employed as linkers to load lipotropic payloads and increase the hydrophilicity [156]. Because the pyrophosphate linker showed high stability in vivo, Kern et al. replaced the traditional Val–Cit–PAB linker with a phosphate diester structure [157]. Recently, payload release based on photoresponsive cleavable linkers has gradually emerged. Photoresponsive linkers incorporate a UV light-controlled O-nitrobenzyl group as a chemical trigger. However, ADCs that undergo cleavage by near-infrared light present challenges including self-aggregation, complex structure and photoinstability, and near-infrared light cannot penetrate skin to reach deeply into the tumor area [158]. There are also bioorthogonally cleavable linkers; for example, although SMCC is a noncleavable linker, studies have identified 2-(maleimidomethyl)-1,3-dioxane (MD) as a potent alternative to the classical SMCC linker because of its greater stability. Another report showed that the use of noncleavable linkers in MMAE-based ADCs could broaden the therapeutic window [159]. Novel chemical triggers have been developed to increase the selectivity of delivery to the tumor area. Developing linkers with simplified structures and integrated functions may be another direction for ADC research.

**Table 5:** Common linkers in drug conjugates

Chemical trigger	Structure	Payload	Characteristics
Noncleavable linker	6-Aminocaproic acid, Transmembrane peptide TAT, Triazole, Oxime, Short peptide, Fragment CGGW, PEG linkers with intermediates of alkyne, triazole and piperazine, Mal-PAB linker	MMAE, PBD Dimer, TRMRA	No linker cleavage. A polypeptide or carbon composed mostly of 4 amino acids, in which the main chain contains 5 to 8 carbon atoms. It is chemically stable and can regulate the polarity of tumor-targeting PDCs [160, 161]
GSH-cleavable linker	Disulfide trigger	DM1, DM3, MMAE	Linker cleavage depends on a threshold level of GSH in the cytoplasm [138, 162]
pH-sensitive linker	Hydrazone, Carbonate, Silyl ether trigger	Calicheamicin, SN-38, MMAE	The linker is not cleaved when entering the circulatory system, but once it reaches the tumor tissue, it is cleaved under the acidic environment and releases the drug [139]
Enzyme-sensitive linker	Glycosidase, Phosphatase, Sulfatase, Dipeptide or tripeptide, Carbamate, Ester and Amide	MMAE, Budesonide DDAE, Paclitaxel	The valine–citrulline (Val–Cit) linker exhibits widespread sensitivity to a variety of cathepsins, thought to be highly expressed in cancer cells, and the widespread sensitivity to other cathepsins could induce off-target toxicity in normal cells [156, 163–166]
Fe(II) cleavable trigger	1,2,4-Trioxolane, PLGLAG, Val–Cit	MMAE, DM1	Linker cleavage is dependent on a threshold level of Fe(II) [155]
Redox-sensitive linker	Disulfide bond	DM1	The disulfide bond is cleaved by glutathione in the tumor tissue, and the cytotoxic load is released
Cathepsin-cleavable linker	Dipeptide trigger, Triglycyl (CX) trigger, cBu-Cit trigger	MMAE, DM1, PBD	Linker cleavage by cathepsin in lysosomes [167]
Phosphatase-cleavable linker	Pyrophosphate trigger	Budesonide	Linker cleavage by phosphatase and pyrophosphate in lysosomes [157]
Sulfatase-cleavable linker	Arylsulfate trigger	MMAE	Linker cleavage by sulfatase in lysosomes [165]
Photoresponsive cleavable linker	Heptamethine cyanine fluorophore trigger, *O*-Nitrobenzyl trigger, PC4AP trigger	CA-4, MMAE, DOX	Linker cleavage by irradiation with NIR light (λ = 650–900 nm), UV light (*λ* = 365 nm) or UV light (*λ* = 365 nm), respectively [158, 168, 169]
Bioorthogonally cleavable linker	dsProc trigger	DOX	Linker cleavage by the bioorthogonal cleavage pair Cu(I)-BTTAA/dsProc [170]

*PDC* peptide drug conjugate, *GSH* glutathione, *MMAE* Monomethyl auristatin E, *PBD* pyrrolobenzodiazepines

#### Drug–antibody ratio (DAR)

The DAR refers to the number of effective payload molecules carried by each antibody and is another important factor related to the activity of the ADC. Once the best linker has been selected for the ADC, it is important to determine the ideal number of conjugates to the antibody. A very low DAR will reduce efficacy, and a high DAR is associated with increased in vitro potency but may also have adverse effects on pharmacokinetic and pharmacological properties [171]. An excess of payloads on a single antibody can destabilize the structure, leading to increased hydrophobicity and toxicity. For example, the binding of doxorubicin or MMAE to an ADC at a high DAR can result in a greater degree of hydrophobicity [172], leading to increased aggregation and a higher clearance rate [173]. This effect can be offset by using hydrophilic linker molecules [174]. The synergistic “1 + 1 > 2” combination of chemotherapy and targeted drug has both increased the treatment efficacy and reduced the incidence of toxic side effects, significantly improving therapeutic outcomes.

The molecular structures of natural antibodies present two main conjugation opportunities, namely, amino conjugation to lysine (Lys) and sulfur conjugation to cysteine (Cys). Since the antibody contains at least 40 modifiable lysine residues and the drug is conjugated randomly to different lysine residues, the conjugation products may contain a complex mixture of many unique molecules. Thus, lysine conjugation results in ADCs with highly variable DARs. The conjugation of cysteine residues may overcome this problem. When all 8 sulfhydryl groups of the antibody react with small molecules, an ADC with a uniform DAR of 8 can be obtained. In theory, the more payloads the ADC carries, the stronger the antitumor effect will be during the treatment window. In reality, however, most ADCs that have been approved or are under clinical development have DARs limited to 24 [175]. In one study, the tubulin inhibitor MMAE was conjugated to the CD30 mAb via cysteine to produce ADCs with different DARs (named E2, E4, and E8, depending on the DAR). The antitumor effects of E2, E4 and E8 were tested in vitro and in vivo, and the results showed that the antitumor efficacy increased with DAR in vitro (IC_50_ E8 < E4 < E2), but E8 had the same antitumor efficacy as E4 in vivo. Thus, the increased DAR did not confer additional efficacy and the maximum tolerated dose (MTD) decreased with increasing DAR (MTD = 50 mg/kg, 100 mg/kg and 250 mg/kg for E8, E4, and E2, respectively) [176]. Pharmacokinetics analysis showed that the clearance rate of the ADC increased with the DAR, which explains why E8 had the same effect as E4 in vivo.

However, the DAR limit can be overcome. A high DAR can facilitate internalization of the ADC, leading to increased efficacy, but it can also increase the clearance rate, resulting in rapid drug elimination. Currently, the DARs of most clinical ADCs are between 2 and 4, which represents a balance between potency and physicochemical properties [177]. However, a recently reported approach, optimizing a cleavable linker molecule with a fleximer scaffold and combining it with an uncleavable linker molecule that is then attached to the antibody, increases the DAR while preserving the pharmacokinetic profile and drug-like properties, thus increasing treatment efficacy at lower antigen expression levels. However, this technology has been evaluated only in vitro and in preclinical models [178]. An optimized cleavable linker based on the GGFG tetrapeptide increased the DAR of T-DXd from 4.1 to 7.7. The extremely wide therapeutic window and high DAR of T-DXd enable the delivery of enough cytotoxic drugs to kill tumor cells with low HER2 expression. There also new platforms to control DARs. Such as hydrophobic (HIC) chromatcolumn- TSKgel HIC-ADC Butyl. The particle size of 5 μm and the hydrophilic nonporous polymer matrix packing is particularly suitable for DAR values of ADC drugs. Antibody deglycosylation of ADC can simplify DAR measurements with rapid DAR analysis within 15 min by deglycosylation processing and LC–MS assays, thus enabling real-time DAR monitoring to optimize the ADC synthesis process. Therefore, when the limitations of linker and conjugation technology are overcome, high DAR benefits cancer patients, and impressive efficacy against low-expression targets is expected to lead to significant changes in clinical practice in the future [179].

#### Resistance

In recent years, ADCs have undergone rapid development in the field of cancer treatment; however, some patients still experience disease progression after receiving ADC treatment, and the problem of drug resistance to ADCs is of increasing concern. Based on the deep understanding of drug resistance mechanisms, the development of novel ADCs and the exploration of combination treatment strategies are particularly important for further increasing the clinical efficacy of ADCs in treating cancer. The mechanisms of drug conjugate resistance are complicated, and possibilities include the following: (1) Antigen-related resistance. Downregulation of target antigen expression on the tumor cell surface prevents ADCs from exerting cytotoxic effects. For example, a decreased expression level of HER2 leads to T-DM1 drug resistance. Similarly, CD30 downregulation leads to drug resistance in anaplastic large cell lymphoma (ALCL) [180]. Thus, dual-epitope ADCs were developed to overcome such resistance (NCT03821233, NCT04695847). Paradoxically, high antigen expression may also reduce ADC effectiveness, possibly through reduced drug exposure. (2) Endocytosis and migration disorders. For optimal efficacy, ADCs must undergo endocytotic uptake by cells. Endocytosis can proceed through different pathways, including clathrin-mediated endocytosis (CME), caveolin (CAV1)-mediated endocytosis, and clathrin caveolin-independent endocytosis. T-DM1 colocalization associated with CAV1 and drug resistance was also demonstrated in an HER2 + cell line [181]. (3) Lysosomal dysfunction: The ADC enters the lysosome, where the cytotoxic drug is released by chemical or enzymatic cleavage. T-DM1 aggregation in the lysosome was observed in cells with long-term exposure to T-DM1 resistance. In such cells, the ADC reaches the lysosomal compartment but has lower proteolytic activity than in sensitive cells, which decreases the activity of lysosomal proteolytic enzymes. Therefore, all ADCs that require degradation by lysosomal acidic proteases, may be subject to this resistance mechanism [182]. (4) Drug efflux pump: A common mechanism of chemoresistance is the elimination of the drug from the cytoplasm by ATP-binding (ABC) transporters [183]. These drug efflux pumps may contribute to resistance to ADCs, as many cytotoxic drugs are substrates of ABC transporters. Multidrug Resistance Gene (MDR1) is a major driver of resistance to Val–Cit–MMAE ADCs, and significantly lower MDR1 activity is observed in AML myeloblasts with a therapeutic response to gemtuzumab ozogamicin than in nonresponders [184]. (5) Mutations in target sites: One potential mechanism of ADC resistance could be cellular target mutations of cytotoxic agents [185]. However, no ADC resistance model with mutations in tubulin, topoisomerase I, or RNA polymerase II has yet been reported. (6) Cell cycle: Cyclin B, which is involved in the G2-M transition, was also recently proposed to be involved in the T-DM1 resistance mechanism. T-DM1 induced an increase in cyclin B levels in T-DM1-sensitive HER2 + breast cancer cells but T-DM1 was not observed in cells resistant to T-DM1. Clinical trials have shown that the antitumor effect of T-DM1 is associated with cyclin B expression, so cyclin B could be used as a biomarker for T-DM1 sensitivity [185]. (7) PI3K/AKT signaling pathway: The activation of PI3K/AKT signaling is correlated with resistance to gemtuzumab ozogamicin in primary AML cells in vitro. The AKT inhibitor MK-2206 significantly increased the sensitivity of resistant cells to gemtuzumab ozogamicin [186]. A clinical trial investigating the safety of T-DM1 in combination with the PI3K inhibitor BYL719 is ongoing (NCT02038010). (8) Apoptosis dysregulation: Changes in the regulation of apoptosis may also regulate sensitivity to ADCs. Overexpression of the antiapoptotic protein BCL-2 is associated with resistance to gemtuzumab ozogamicin [187]. High expression of BCL-XL is also associated with reduced sensitivity to CD79b–Val–Cit-MMAE [188]. The administration of a BCL-2 family inhibitor increases ADC activity in vivo [189].

Based on the aforementioned resistance mechanisms, resistance to the antibody components of ADC can be conferred by downregulation or mutation of the target cell surface antigen, and resistance to payload toxicity can be conferred by increased drug efflux transporter activity. Unique resistance mechanisms specific to the mode of action of ADCs have also emerged, such as altered internalization or cell surface recycling of targeted tumor antigens, changes in the intracellular routing or processing of ADCs, and impaired release of toxic payloads into the cytoplasm. Combination therapies are more promising than single-agent therapies for overcoming drug resistance. FDA-approved ADCs provide valuable treatment options for difficult-to-treat patient populations, but drug resistance is a frequently encountered limitation, and appropriate combination therapies may increase the percentage of cancer patients who receive long-term therapeutic benefits [190].

## Antibody–drug conjugates (ADCs)

**Fig. 3:**
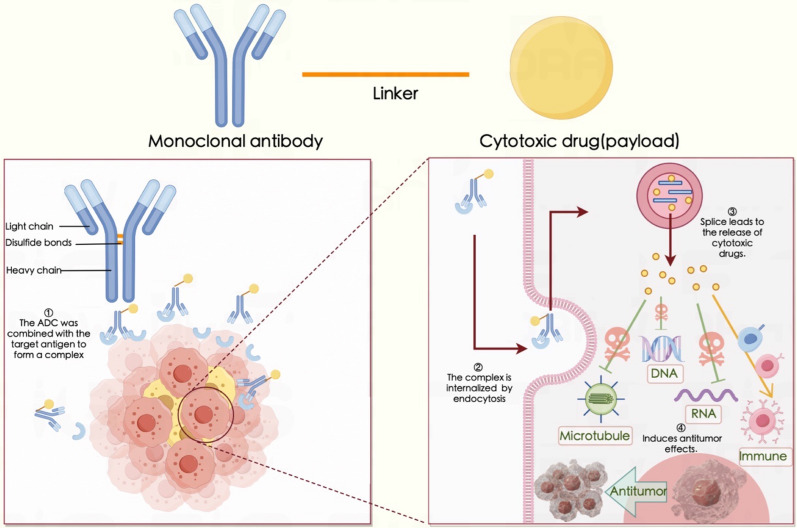
Mechanism of antibody–drug conjugates. ADCs bind to target antigens to form complexes, which are internalized by endocytosis. Linker cleavage leads to the release of cytotoxic drugs. *ADC* antibody‒drug conjugate

ADCs are a relatively new class of anticancer drugs [191] designed to combine the target selectivity of monoclonal antibodies with the cytotoxic properties of chemotherapeutic drugs [192]. Chemotherapy is still one of the main methods of cancer treatment, and many chemotherapeutic drugs are widely used in clinical practice; however, many adverse effects (AEs) and drug resistance problems are associated with these methods. ADCs can directly deliver cytotoxic drugs to tumor sites, transforming chemotherapy into targeted therapy. ADCs contain three essential factors: antibodies targeting specific tumor antigens, cytotoxic drugs (also known as payloads or warheads) and linkers connecting the payloads to the antibodies [32]. After an ADC enters the circulatory system, it combines with the target antigen to form a complex [193, 194]. The complex is internalized by endocytosis [195, 196], and cleavage of the linker leads to the release of the cytotoxic drug [197, 198] (Fig. 3). The antibody component can specifically recognize tumor antigens expressed at the target site, and the linker acts as a bridge to carry cytotoxic small molecules with significant lethal effects [199]. This approach combines mAb drugs and small-molecule chemical drugs, utilizing antibodies to achieve tumor targeting and efficiently eliminating tumor cells by releasing cytotoxins with strong killing effects in the target tissues. Advantages such as high activity, low toxicity and a long duration of action have allowed ADCs to greatly increase the therapeutic indices of small-molecule chemical drugs. Moreover, this approach partially solves the problems of low activity and high drug resistance associated with mAb drugs.

After the development of three generations of ADCs, they are considered a mature technology. Mylotarg, which targets CD33, is a representative first-generation ADC [200]. Mouse-derived antibodies have strong immunogenicity and are prone to inducing the production of human anti-mouse antibodies. The linker is unstable, and the toxin is quickly released into the plasma, leading to severe toxic side effects. However, the efficacy of the cytotoxic drugs is insufficient for killing tumor cells [201]. Second-generation ADCs are represented by Adcetris, which targets CD30, and Kadcyla, which targets HER2: in these ADCs, human–mouse chimeric antibodies and humanized monoclonal antibodies were used instead of mouse-derived monoclonal antibodies, along with more cytotoxic payloads and more stable linkers [202, 203]. However, the DARs are uneven [204, 205], and naked antibodies that are not bound to cytotoxic moieties enter the circulation, where they compete for conjugate antigen binding sites and reduce efficacy. In addition, the binding of excessive drug molecules to antibodies can easily cause problems such as antibody aggregation, accelerated clearance, and increased nonspecific toxicity [206, 207]. For third-generation ADCs, due to the development of fixed-point conjugation technology, DARs have been stabilized at approximately 2–4, and the stability and pharmacokinetic properties have improved [208, 209]. More hydrophilic linker modifications, such as PEGylation, are also employed in the third generation of ADCs [210, 211]. Moreover, the bystander effect [212, 213], which is achieved by the use of cleavable connectors, increases treatment efficacy and reduces systemic toxicity. A representative example is Enhertu, which targets HER2.

### Target and antibody selection

The ideal target for an ADC is an antigen that is expressed only on the surface of tumor cells [214]. Targets that are preferentially expressed in tumors compared to nonmalignant tissues have a wider therapeutic window and a lower likelihood of systemic toxicity [215]. Therefore, choosing the appropriate antigen is one of the major challenges in the development of ADCs. Based on this requirement, three aspects should be considered in antigen selection: (1) high expression in tumors and low expression in healthy tissues [216]; (2) expression of the target antigen on the surface of tumor cells, making it accessible to antibodies; and (3) the existence of a pathway of intracellular transportation and a suitable internalization rate. Notably, noninternalizing ADCs can also exert therapeutic effects through an alternative “bystander effect” [217], in which a membrane-permeable drug can induce the death of neighboring cells [218].

A suitable antibody should have high target specificity, abundant target expression, and an appropriate internalization rate [219]. Due to problems such as acute hypersensitivity reactions and the side effects of neutralizing antibodies when murine antibodies are used, the antibodies currently used in ADCs are mainly humanized antibodies, which have significantly lower immunogenicity than murine and chimeric monoclonal antibodies. They also have higher solubility and a longer half-life. Nonetheless, although the use of humanized antibodies can minimize the problems encountered with mouse-derived antibodies, these problems are not completely solved [220]. Most antibodies used in clinical practice are derived from human immunoglobulin (IgG), which has a molecular weight of approximately 150 kDa and consists of two heavy chains and two light chains. Antibody derivatives can generally be divided into antigen-binding fragments (Fabs), single-chain variable fragments (scFvs) and variable domains (VHHs). Fabs and scFvs retain the size and affinity of the antigen-binding region and are smaller than conventional IgG [221], resulting in improved the pharmacokinetic properties for tumor penetration [222, 223]. Antibodies also need to bind to antigens with appropriate affinity to increase accumulation and prolong the retention time at the tumor site. However, if the retention time is too long, the paracancerous cells surrounding the solid tumor may be compromised [224].

### ADCs targeting HER2/HER3

Human epidermal growth factor receptor 2 (HER2) is a receptor tyrosine kinase encoded by the ERB-B2 receptor tyrosine kinase 2 (ERBB2) gene. The HER family consists of four members: HER1 (EGFR/ErbB1), HER2, HER3 (ERBB3) and HER4 (ERBB4) [225, 226]. The HER family, especially HER2, is considered a therapeutic target in lung cancer because it is overexpressed or mutated in multiple tumors, including lung cancer, and the activation of related pathways, such as MAPK, PI3K, AKT and PKC, can lead to excessive cell proliferation [227], resulting in tumor occurrence and development. In addition to corresponding targeted therapies, ADCs can exhibit excellent antitumor activity by acting on the HER family.

#### T-DM1

T-DM1 consists of the HER2 monoclonal antibody trastuzumab and the microtubule inhibitor emtansine (DM1) linked via a nonreducible sulfur linker, with an average of 3.5 payload molecules per antibody [228, 229]. T-DM1 was the first ADC to be tested against advanced HER2-positive NSCLC and provides a new treatment strategy for patients with advanced HER2-positive disease [230]. A phase II clinical trial including 18 patients with advanced HER2-mutated NSCLC showed a partial response (PR) in 8 patients, with a median progression-free survival (PFS) of 5 months [231]. Another phase II study reported similar results, with an objective response rate (ORR) of 51% and a median PFS of 5 months [232] in 49 patients with HER2 mutation or overexpression. Based on these data, the National Comprehensive Cancer Network (NCCN) recommends T-DM1 as a class 2A drug for the treatment of advanced HER2-mutated NSCLC [233, 234]. However, two other phase II clinical studies showed limited efficacy of T-DM1 in HER2-positive or HER2-overexpressing NSCLC patients. In preclinical studies of HER2-immunohistochemistry score 3 + CALU-3 lung cancer cells, T-DM1 dose-dependently inhibited tumor cell growth. A phase I study investigating HER2 overexpression in 49 previously treated patients with advanced NSCLC reported ORRs of 0% and 20% for HER2 IHC2 + and 3 + , respectively, with median PFS times of 2.6 and 2.7 months [234]. In addition, T-DM1 has shown significant efficacy in the treatment of lung cancer with HER2 exon 20 insertions [235]. The main adverse effects of T-DM1 include transaminase elevation, thrombocytopenia and nausea [231].

#### T-DXd

T-DXd (trastuzumab deruxtecan), also known as DS-8201, is a novel HER2-targeting ADC [236] with a different mechanism of action from that of other ADCs: it binds and stabilizes topoisomerase I-DNA complexes, inducing DNA double-strand breaks and apoptosis [237]. T-DXd consists of trastuzumab, a cleavable linker, and the topoisomerase I inhibitor deruxtecan [238]. T-DXd has satisfactory membrane permeability and can not only kill HER2-positive tumor cells but also exert bystander effects to kill nearby tumor cell [239]. T-DXd has a high DAR of 8, indicating that an average of 8 effective payload molecules can be conjugated to each trastuzumab molecule [240]. T-DXd has shown good antitumor activity in patients with HER2-mutated solid tumors (except for breast and gastric cancer). The latest data showed an overall ORR of 72.7% and a median PFS of 11.3 months in 11 previously treated NSCLC patients with HER2 mutation [241]. Subsequently, an open-label, global phase II DESTINY-Lung01 clinical study was conducted for advanced NSCLC patients with HER2 overexpression or mutation. Among 91 patients with HER2-mutated NSCLC, the ORR was 55%, the disease control rate (DCR) was 92%, the median PFS was 8.2 months, and the median OS was 17.8 months [242]. In January 2021, the World Conference on Lung Cancer (WCLC) released data on HER2-overexpressing NSCLC patients treated with T-DXd. Among the 49 patients, the ORR was 24.5%, the DCR was 69%, and the median PFS was 5.4 months. Subgroup analysis revealed that the ORRs of the IHC3 + and IHC2 + groups were 20% and 25.6%, respectively, indicating that HER2 IHC expression had no significant effect on the ORR. Currently, the phase Ib DESTINY-Lung03 clinical study exploring the clinical efficacy of T-DXd combined with durvalumab and chemotherapy in newly diagnosed HER2-positive advanced NSCLC patients is ongoing. Therefore, T-DXd is more effective for treating advanced NSCLC with HER2 mutations. Regarding toxicity, the most common adverse reactions to T-DXd are gastrointestinal and hematologic toxicities, with neutropenia being the most common grade 3 adverse reaction [243]. Importantly, interstitial lung disease (ILD) was observed in 11.9% of HER2 mutation patients (all grade 2), with a median onset time of 86 days [244]. The incidence of ILD was slightly greater in the HER2-overexpressing population (16.3%), which included 3 patients with grade 5 ILD. Overall, T-DXd has good overall safety, but patients treated with T-DXd need to be closely monitored for the occurrence of ILD.

#### A166 and MRG003

A166 is an ADC targeting HER2 that consists of a microtubule inhibitor connected to trastuzumab via a cleavable linker. Data from a phase I clinical study evaluating A166 in 81 patients with advanced solid tumors showed an ORR greater than 60%. Regarding safety, the most common adverse reactions include keratitis, dry eye, blurred vision and decreased appetite [245].

MRG003 is a novel ADC targeting EGFR that showed remarkable preliminary efficacy in phase I clinical studies for various solid tumors. Clinical trials of MRG003 for late-stage EGFR-mutant NSCLC are still ongoing, and MRG003 is expected to become China’s first anti-EGFR ADC.

#### U3-1402

U3-1402 (patritumab deruxtecan) is an ADC targeting HER3, another member of the EGFR family, and consists of a humanized anti-HER3 antibody and a topoisomerase I inhibitor payload [246]. HER3 is overexpressed in 19% of NSCLCs and up to 46% of adenocarcinomas [247] and is involved in mediating resistance to EGFR tyrosine kinase inhibitors (TKIs) [248]. A phase I study enrolled 57 patients with advanced NSCLC without the T790M mutation who progressed after EGFR-TKI treatment, and almost all the patients were found to express HER3. The results showed that the ORR of U3-1402 monotherapy was 39%, with a median PFS of 8.2 months, suggesting that U3-1402 could be an important treatment option for patients with NSCLC with multidrug resistance [249]. At American Society of Clinical Oncology (ASCO) 2021, the latest data from a phase I dose escalation/expansion trial including 39 patients with locally advanced or metastatic NSCLC with EGFR mutations who had experienced disease progression after EGFR-TKI therapy were presented. The ORR was 39%, and the DCR was 72%. With a median follow-up of 10.2 months, the median DoR was 6.9 months, and the median PFS was 8.2 months [249]. Subgroup analyses also revealed the antitumor activity of U3-1402 in NSCLC patients harboring resistant EGFR mutations. A phase II study of U3-1402 is ongoing. U3-1402 lacks significant efficacy compared to that of other targeted drugs but may be a new treatment option for patients who are resistant to third-generation TKIs or who are otherwise not suitable for third-generation TKI treatment. In terms of safety, 47% of patients experienced grade 3 or higher adverse events, among which thrombocytopenia (28%) and neutropenia (19%) were the most common.

### ADCs targeting Trop-2

Trophoblast cell surface antigen 2 (Trop-2) is a transmembrane protein that is closely related to cell proliferation and differentiation. Trop-2 is expressed at low or almost undetectable levels in normal tissues and overexpressed in various epithelial cancers, including NSCLC and SCLC. Trop-2 overexpression has also been shown to be associated with poor prognosis in lung adenocarcinoma [250], suggesting that Trop-2 has potential as a new target for lung cancer treatment.

#### DS-1062a

DS-1062a (datopotamab deruxtecan) is an ADC in which a Trop-2-targeting antibody is connected to a topoisomerase I inhibitor payload via a tetrapeptide linker. DS-1062a may have antitumor effects on multiple types of cancer. The latest research results on the tolerability and safety of DS-1062a in treating advanced NSCLC in a clinical trial were presented at the 2021 ASCO Annual Meeting. In NSCLC patients receiving different 4.0, 6.0, and 8.0 mg/kg doses of DS-1062a, the ORRs were 31%, 20%, and 26.3%, respectively, and the DCRs were 79%, 75%, and 79%, indicating that DS-1062a has good antitumor activity in lung cancer. In another phase I study of 175 recurrent/refractory advanced NSCLC patients, the ORRs of DS-1062a at doses of 4, 6, and 8 mg/kg were 23%, 21%, and 25%, respectively, and the median PFS times were 4.3, 8.2, and 5.4 months, respectively. Treatment-related adverse reactions were dose dependent, with a grade ≥ 3 incidence of 10–34%, and included oral mucositis, nausea, fatigue, mucositis, and anemia. Among them, four patients in the 8 mg/kg group experienced grade ≥ 3 ILD. The most common grade ≥ 3 adverse reactions in patients treated with DS-1062a at different doses were oral mucositis, mucosal inflammation, nausea, fatigue, and anemia.

#### IMMU-132

IMMU-132 (sacituzumab govitecan) is an ADC in which the topoisomerase I inhibitor SN-38 (the active metabolite of irinotecan) is linked to a humanized anti-Trop-2 antibody via a cleavable linker with a DAR of 7.6 [251]. In a phase I clinical trial including 25 patients with standard therapy-refractory metastatic solid tumors (including NSCLC and SCLC), 2 patients achieved PR, and 16 patients had stable disease [252]. Based on these results, the trial entered a phase II exploration with a total of 495 patients enrolled. The researchers evaluated 54 patients with advanced NSCLC, with an ORR of 16.7%, a median DoR of 6.0 months, a median PFS of 4.4 months, and a median OS of 7.3 months. In another group of 62 patients with first-line chemotherapy-resistant or sensitive metastatic SCLC, the ORR was 17.7%, the median DoR was 5.7 months, the median PFS was 3.7 months, and the median OS was 7.1 months [253]. Currently, IMMU-132 is undergoing phase Ib/II clinical trials in combination with atezolizumab for NSCLC and in combination with the ATR inhibitor berzosertib for SCLC. Regarding toxicity, grade 3 adverse events included diarrhea, fatigue, anemia, nausea, and neutropenia [254]. The data above suggest that IMMU-132 may be a promising drug for treating NSCLC and SCLC.

#### Dato-DXd

Dato-DXd has been explored for use in lung cancer treatment and has shown broad application prospects [255]. The TROPION-Lung02 trial is an ongoing global, open cohort phase Ib study to evaluate the safety and effectiveness of Dato-DXd (4 or 6 mg/kg) + pembrolizumab (200 mg) ± platinum chemotherapy (cisplatin or carboplatin) in patients with advanced or metastatic NSCLC who are newly treated or previously treated and who have no driver gene mutations. The median follow-up times for the dual drug group and the triple drug group were 6.5 months and 4.4 months, respectively, at which times 53% and 77%, respectively, of patients in the two groups were still receiving treatment. The median treatment durations were 4.1 months and 3.0 months, respectively. In the first-line treatment groups, the ORRs in the dual drug group and the triple drug group were 62% and 50%, respectively, and the disease control rates were 100% and 90%, respectively. In the second-line treatment groups, the ORRs were 24% and 29%, respectively. This combination regimen is well tolerated and exhibits encouraging antitumor activity as a first-line treatment.

### ADCs targeting c-Met

The c-Met protein is encoded by the gene mesenchymal-epithelial transition (Met) and is a tyrosine kinase receptor expressed on the surface of both epithelial and endothelial cells [256–258]. When activated, it promotes cell proliferation, growth, migration, and angiogenesis. The abnormal activation of the c-Met pathway in NSCLC mainly involves Met14 exon skipping mutations, Met fusion and overexpression, and MET amplification, which is also a resistance mechanism in EGFR-mutant NSCLC resistant to EGFR TKIs [259]. The incidence of MET14 exon skipping mutation is 3–4%, the incidence of primary MET amplification is approximately 3%, and the incidence of secondary amplification is 10–15%; MET amplification is associated with resistance to multiple TKIs, and the incidence of overexpression, which is a predictor of poor prognosis, is approximately 24% [244, 260–262].

#### Teliso-V

Teliso-V is an ADC composed of an anti-MET monoclonal antibody (ABT-700) linked to the cytotoxic payload MMAE, which inhibits microtubule polymerization. The key to the mechanism of action is that after antibody binding, the cytotoxic payload can be directly delivered to tumor cells, limiting potential resistance mechanisms related to intracellular signaling, such as ME3 amplification in EGFR TKI resistance. A phase I study showed that Teliso-V, either as a single agent or in combination with erlotinib, was well tolerated in patients with advanced MET-positive NSCLC and exhibited good antitumor activity both as a monotherapy and in combination with erlotinib. In a separate phase I dose escalation and expansion study, Teliso-V was shown to be effective as a single agent only in MET-positive advanced NSCLC patients [263]. However, a phase II study evaluating the efficacy of Teliso-V in patients with MET-positive advanced squamous cell NSCLC was terminated early due to severe adverse reactions and a low ORR [264]. Recently, targeted therapy has shown good antitumor activity in patients with Met14 exon skipping mutations, but there is no standard treatment that addresses Met amplification.

#### ABBV-399

ABBV-399 (telisotuzumab vedotin) consists of the microtubule inhibitor MMAE conjugated to a humanized anti-c-Met monoclonal antibody via a cleavable linker with a DAR of 3.1 [265]. A phase I study of 58 patients with advanced c-Met-positive NSCLC showed an ORR of 18.8%, a median DoR of 4.8 months, and a median PFS of 5.7 months [263]. Based on these encouraging results, the phase II trial SWOG S1400K was designed to evaluate the efficacy of ABBV-399 in 23 patients with c-Met-positive advanced squamous NSCLC, but the study was terminated early due to a lack of expected results [264]. Another phase II trial including 52 patients with c-Met-positive NSCLC showed that 9 patients (23%) achieved objective responses, with a median DoR of 8.7 months and a median PFS of 5.2 months [266]. ABBV-399 showed promising efficacy against nonsquamous NSCLC in a phase II study, with an ORR of 35.1% in patients with c-MET-positive, wild-type EGFR nonsquamous NSCLC, 53.8% in the high-expression group and 25% in the moderate expression group; however, ABBV-399 had only limited efficacy in the patient groups with EGFR mutation and squamous NSCLC [264]. Overall, ABBV-399 has shown encouraging efficacy in treating relapsed/refractory nonsquamous NSCLC with c-MET overexpression and wild-type EGFR, but further studies will be needed to validate its efficacy in patients with squamous NSCLC and EGFR-mutant NSCLC.

### ADCs targeting DLL3

Delta-like protein 3 (DLL3) is a ligand that inhibits the Notch signaling pathway, which is involved in multiple processes associated with growth and development. DLL3 is highly expressed in 72% of primary SCLC tumor tissues and 85% of recurrent SCLC tumor tissues [267], whereas it is rarely expressed in normal tissues, making it a promising target [268, 269].

#### Rova-T

Rova-T (rovalpituzumab tesirine) is an ADC that targets DLL3 and consists of an anti-DLL3 monoclonal antibody, a DNA-damaging pyrrolobenzodiazepine dimer toxin, and a protease-cleavable linker [270]. In a phase I clinical trial, the ORR of 74 recurrent SCLC patients treated with Rova-T was 18%, with a median PFS of 3.1 months and a median OS of 4.6 months [271]. The TRINITY study was a phase II trial in which Rova-T was applied as a third-line treatment to 339 patients with DLL3-expressing SCLC; the ORR was 12.4%, the median PFS was 3.5 months, and the median OS was 5.6 months [272]. The TAHOE study compared the efficacy of Rova-T and topotecan as second-line treatments for SCLC [273]. The Rova-T group and the topotecan group included 296 and 148 patients, respectively. The results showed that the median PFS and OS in the Rova-T group were 3.0 months and 6.3 months, respectively, while they were 4.3 months and 8.6 months in the topotecan group. Because the PFS and OS of the Rova-T group were both worse, the study was terminated early. Another phase III MERU study was likewise terminated early due to limited efficacy [244]. Based on the results of monotherapy, a phase I/II clinical trial explored the efficacy of Rova-T in combination with nivolumab or in combination with both nivolumab and ipilimumab in 42 patients with advanced-stage SCLC; the resulting ORR was 30%, the median PFS was 4.2 months, and the median OS was 7.4 months [274]. Another phase I study evaluated the efficacy of Rova-T in combination with budesonide in 31 SCLC patients, and the ORR was 24.1% [275]. Overall, Rova-T monotherapy has limited benefits for SCLC patients, but combination therapy is expected to be effective. In terms of safety, 38 to 64% of patients experienced grade 3 or higher adverse reactions, among which the most common were platelet count reduction, pleural effusion, and elevated lipase [276].

#### SC-002

SC-002 is an ADC composed of a humanized anti-DLL3 monoclonal antibody linked to SC-DR002 by a cleavable linker, with a DAR of 2 [277]. Phase I studies included 35 patients with relapsed/refractory SCLC or large-cell neuroendocrine tumors, and the ORR was only 14% (5/35); for DLL3-positive patients, the ORR was only 11.8%. Overall, ADCs targeting DLL3 have proven to be unsuccessful.

### ADCs targeting AXL

AXL is a receptor tyrosine kinase that promotes tumor development through multiple pathways and is associated with chemotherapy and immune therapy resistance in various types of cancer [278]. In NSCLC, AXL activation is associated with EGFR-targeted therapy resistance and lower survival rates in patients with advanced NSCLC [279]. Therefore, AXL is an attractive target for antitumor therapy. Enav (enapotamab vedotin) and BA3011 are ADCs that target this pathway. Enav consists of an anti-AXL monoclonal antibody linked to the microtubule inhibitor MMAE via a cleavable linker [280]. The most common grade 3 or higher adverse reactions observed were gastrointestinal reactions, which included constipation, colitis, diarrhea, bloating, nausea, and vomiting. However, because of the low efficacy, the clinical development was terminated.

### ADCs targeting NaPi2b

NaPi2b is a sodium-dependent phosphate transporter encoded by SLC34A2 that has been shown to play a role in cell differentiation and tumorigenesis [281]. NaPi2b is highly expressed in various cancers, including lung cancer, particularly in patients who are TTF1-positive or have KRAS and EGFR mutations [282]. Because of its elevated expression in multiple cancers, NaPi2b is an attractive target for ADC development.

XMT-1536 is an ADC composed of a humanized anti-NaPi2b targeting antibody and the potent payload auristatin F-hydroxypropylamide (AF-HPA). A preclinical study showed that XMT-1536 had strong antitumor efficacy in mouse models of NSCLC and ovarian cancer [283]. Phase I/II dose escalation and expansion studies of XMT-1536 for the treatment of refractory advanced NSCLC are still ongoing.

### ADCs targeting CEACAM5

CEACAM5, also known as CD66e, is a glycoprotein encoded by the carcinoembryonic antigen gene and is expressed at low levels in normal tissues but at moderate to high levels in multiple cancers, including NSCLC: 20% of nonsquamous NSCLCs exhibit high expression (> 50%), and 25% exhibit moderate expression (1–49%) [213]. SAR408701 is a novel ADC composed of a humanized anti-CEACAM5 monoclonal antibody and the microtubule inhibitor maytansinoid DM4, connected by a cleavable tetrapeptide linker, with a DAR of 3.9. In the first clinical study, which included 92 patients with advanced NSCLC for whom previous treatments had failed, SAR408701 achieved ORRs of only 7.1% in the moderate CEACAM5 expression group and 20.3% in the high CEACAM5 expression group, with a median DOR of 5.6 months. The incidence of ≥ 3 AEs was 47.8%, of which 15.2% were drug related, including keratitis (10.9%) and fatigue (4.3%). The most severe AE was dyspnea related to disease progression. A phase III clinical trial (NCT02187848) investigating combined first-line chemotherapy and immunotherapy for advanced NSCLC patients with high CEACAM5 expression is currently underway, with hopes of clinical benefit.

Antibodies play a crucial role in the internalization of ADCs into tumor cells. Therefore, identifying antibodies with high specificity and affinity for the target antigen is essential. Dual-targeting antibodies can not only increase internalization but also increase the specificity for tumor cells. Compared to single-targeting antibodies, dual-targeting antibodies have higher antitumor activity and may be a useful new research direction [284]. Additionally, converting traditional antibody frameworks into “small” peptide fragments or single-chain variable fragments can increase tissue permeability and payload transmission by reducing the molecular weight of conjugates [285]. Moreover, innovative payloads can contribute to improving the antitumor effects of ADCs. Increasing the DAR is another important method for improving the antitumor efficacy of ADCs. Preclinical studies have shown that dolaflexin technology can increase the DAR and thereby induce tumor regression [178].

### Other targets in the development of ADCs for lung cancer treatment

**Fig. 4:**
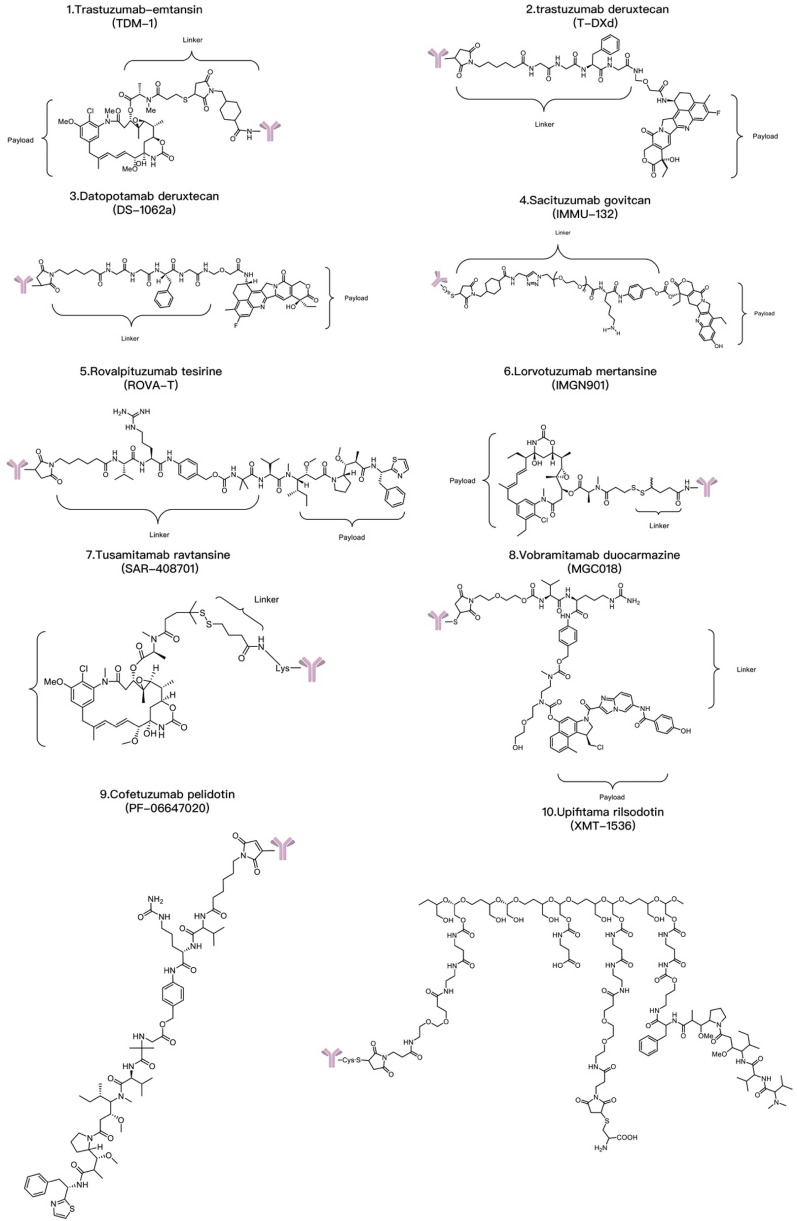

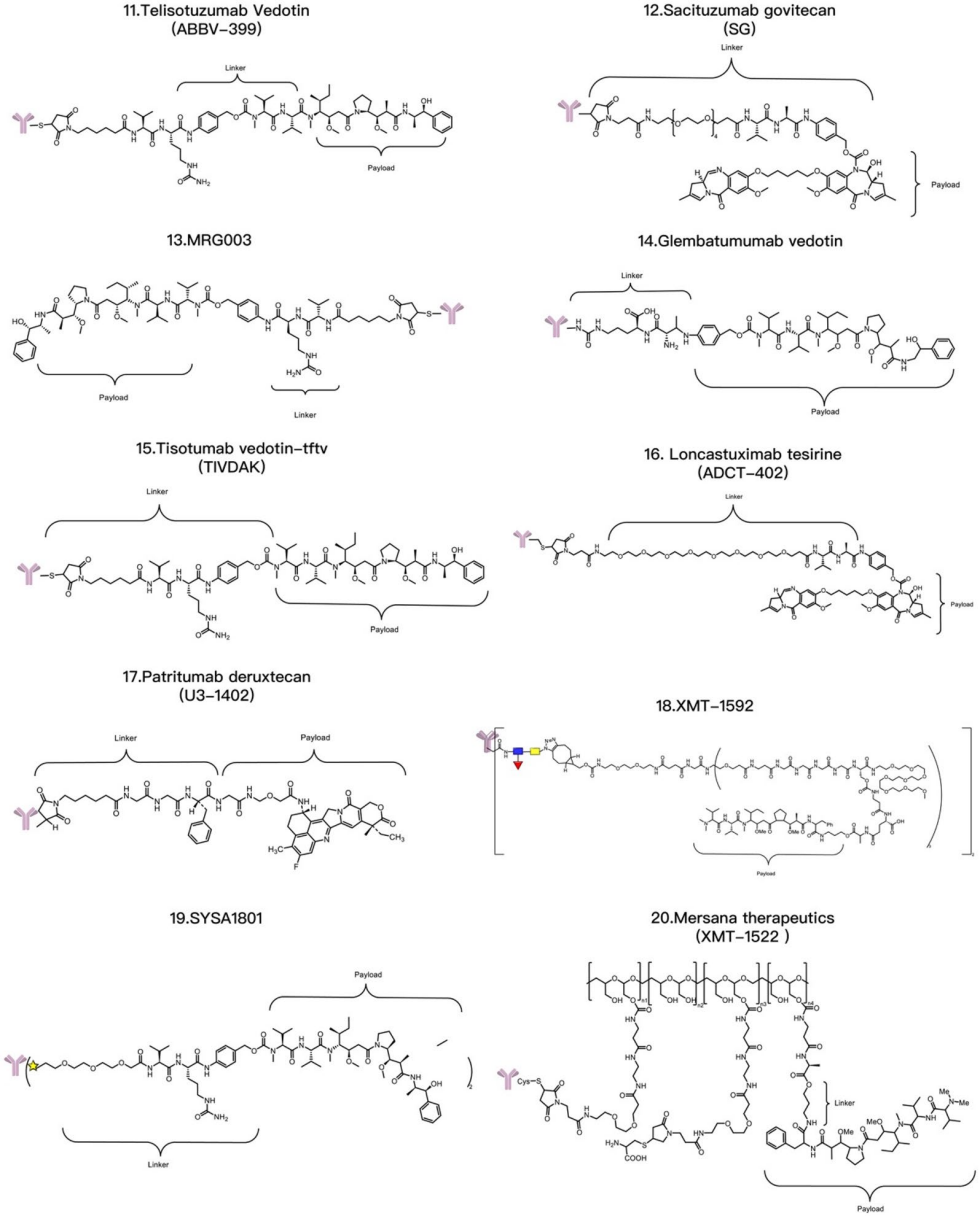
Chemical structures of representative antibody‒drug conjugates (ADCs) in clinical trials for lung cancer treatment

Different ADCs targeting other transmembrane proteins or membrane receptors, including CD19, TF, PTK7, and B7-H3, are currently undergoing clinical trials for lung cancer [286] (Table 6). The composition of each ADC is shown in Table 7, and the chemical structure of each ADC is shown in Fig. 4.

**Table 6:** Clinical trials of ADCs in lung cancer

ADC drug name	Target	NCT number	Status (efficacy)	Study phase	Number of subjects	Primary endpoint	Start date
T-DM1	HER2	NCT04591431	Not recruiting	II	384	ORR	Oct 2020
T-DXd	HER2	NCT04686305	Recruiting	Ib	136	ORR; PFS; OS	Mar 2021
T-DXd	HER2	NCT05048797	Recruiting	III	246	PFS	Oct 2021
T-DXd	HER2	NCT05246514	Not recruiting	II	66	ORR	Jul 2022
T-DXd	HER2	NCT05650879	Recruiting	Ia/Ib	178	DLT, AEs	Mar 2023
T-DXd	HER2	NCT05091528	Terminated	I/II	2	DLT, AEs	Feb 2022
T-DXd	HER2	NCT04644068	Recruiting	I/II	559	AEs	Nov 2020
T-DXd	HER2	NCT03505710	Not recruiting	II	181	ORR	May 2018
T-DXd	HER2	NCT04042701	Recruiting	I	115	AEs	Feb 2020
T-DXd	HER2	NCT05048797	Recruiting	III	264	PFS	Oct 2021
T-DXd	HER2	NCT04686305	Recruiting	I	136	ORR; PFS; OS	Mar 2021
T-DXd	HER2	NCT05246514	Not recruiting	II	66	ORR	Jul 2022
T-DXd	HER2	NCT04644237	Not recruiting	II	152	ORR	Mar 2021
T-DXd	HER2	NCT03334617	Recruiting	II	570	ORR; PFS; OS	Dec 2017
XMT-1522	HER2	NCT02952729	Completed (NA)	I	120	Time of maximum concentration	Nov 2016
ADCT-402	CD19	NCT04235101	Completed (NA)	I	120	ORR	Apr 2023
ADCT-402	CD19	NCT02277717	Completed (NA)	I	185	AEs	Jan 2019
U3-1402	HER3	NCT04619004	Not recruiting	II	420 [249]	ORR; PFS; OS	Feb 2021
U3-1402	HER3	NCT04676477	Recruiting	I	252 [249]	ORR; PFS; OS	Jun 2021
U3-1402	HER3	NCT05338970	Recruiting	III	560	PFS	May 2022
SG	Trop-2	NCT05119907	Recruiting	II	300	DOR; PFS; OS	Oct 2021
DS-1062a	Trop-2	NCT04484142	Not recruiting	II	137	ORR	Mar 2021
DS-1062a	Trop-2	NCT04940325	Recruiting	II	100	ORR	May 2021
DS-1062a	Trop-2	NCT04656652	Not recruiting	III	590	PFS; OS	Dec 2020
DS-1062a	Trop-2	NCT05460273	Not recruiting	I/II	118	ORR	Jul 2022
DS-1062a	Trop-2	NCT03401385	Recruiting	I	770	DLT, AEs	Jan 2018
DS-1062a	Trop-2	NCT05555732	Recruiting	III	975	PFS; OS	Jan 2023
DS-1062a	Trop-2	NCT04526691	Not recruiting	I	140	DLT, AEs	Sep 2020
DS-1062a	Trop-2	NCT03944772	Recruiting	II	250	ORR	Jun 2019
DS-1062a	Trop-2	NCT04612751	Recruiting	Ib	232	DLT, AEs	Feb 2021
IMMU-132	Trop-2	NCT05089734	Not recruiting	III	580	OS	Nov 2021
IMMU-132	Trop-2	NCT05186974	Recruiting	II	224	DLT	May 2022
IMMU-132	Trop-2	NCT04826341	Recruiting	I/II	85	TLT, ORR	Sep 2021
IMMU-132	Trop-2	NCT05609968	Recruiting	III	614	PFS; OS	Feb 2023
IMMU-132	Trop-2	NCT01631552	Completed (Positive)	I/II	515	ORR	Dec 2012
IMMU-132	Trop-2	NCT03337698	Recruiting	Ib/II	435	ORR	Jan 2018
IMMU-132	Trop-2	NCT05627960	Recruiting	I	77	MTD; MAD; ORR	Feb 2022
Skb-264	Trop-2	NCT05631262	Not yet recruiting	II	0	PFS, OS	Nov 2022
Skb-264	Trop-2	NCT05870319	Not yet recruiting	III	0	PFS	Jun 2023
Skb-264	Trop-2	NCT05816252	Recruiting	II	296	ORR	Apr 2023
Skb-264	Trop-2	NCT05351788	Recruiting	II	110	AEs	Apr 2022
Dato-DXd	Trop-2	NCT05215340	Recruiting	III	740	PFS; OS	Mar 2022
Dato-DXd	Trop-2	NCT04526691	Not recruiting	I	145	DLT	Sep 2020
ABBV-399	c-MET	NCT03539536	Recruiting	II	275	ORR; AEs	Oct 2018
ABBV-399	c-MET	NCT04928846	Recruiting	III	698	PFS	Mar 2022
ABBV-399	c-MET	NCT03574753	Completed	II	28	ORR	Mar 2018
ABBV-399	c-MET	NCT05513703	Recruiting	II	70	ORR	Nov 2022
MYTX-011	c-MET	NCT05652868	Recruiting	I	150	ORR; PFS; OS	Mar 2023
Teliso-V	c-MET	NCT04928846	Recruiting	III	698	PFS; OS	Mar 2022
Rova-T	DLL3	NCT03061812	Completed (Negative)	III	444	ORR; OS	Apr 2017
Rova-T	DLL3	NCT03033511	Terminated	III	748	PFS; OS	Feb 2017
Rova-T	DLL3	NCT03334487	Withdrawn	III	0	PFS; OS	Mar 2018
Rova-T	DLL3	NCT03543358	Completed (Negative)	II	3	SAEs	Sep 2018
Rova-T	DLL3	NCT02674568	Completed (Negative)	II	342	ORR; OS	Jan 2016
Rova-T	DLL3	NCT03026166	Terminated	I/II	42	ORR; PFS; OS	Mar 2017
Rova-T	DLL3	NCT02709889	Terminated	I/II	200	ORR; PFS; OS	Sep 2016
Rova-T	DLL3	NCT01901653	Completed (Positive)	I/II	82	ORR; DLT	Jul 2013
Rova-T	DLL3	NCT03086239	Completed (NA)	I	29	ORR; PFS; OS	Apr 2017
Rova-T	DLL3	NCT02874664	Completed (NA)	I	46	AEs	Sep 2016
Rova-T	DLL3	NCT02819999	Terminated	I	28	DLT; TEAEs	Oct 2016
ABBV-181	DLL3	NCT03000257	Completed (NA)	I	182	MTD	Dec 2016
BA3011	AXL	NCT04681131	Recruiting	II	240	ORR	Mar 2021
BA3011	AXL	NCT03425279	Recruiting	I/II	120	ORR	Feb 2018
AXL-107-MMAE	AXL	NCT02988817	Completed (Negative)	I/II	306	DLTs	Nov 2016
TIVDAK	TF	NCT03245736	Completed (Negative)	II	5	AEs	Aug 2017
TIVDAK	TF	NCT02552121	Completed (Negative)	I/II	33	AEs	Nov 2015
TIVDAK	TF	NCT02001623	Completed (Positive)	I/II	195	AEs	Nov 2013
TIVDAK	TF	NCT03485209	Recruiting	II	532	ORR	Jun 2018
Tisotumab vedotin	TF	NCT03913741	Completed (NA)	I/II	23	AEs	Aug 2020
PF-06647020	PTK7	NCT02222922	Completed (Positive)	I	138	DLT	Oct 2014
SAR408701	CEACAM5	NCT04154956	Recruiting	III	450	PFS; OS	Jan 2017
SAR408701	CEACAM5	NCT04394624	Recruiting	II	43	ORR	Aug 2020
SAR408701	CEACAM5	NCT04524689	Recruiting	II	120	ORR	Oct 2020
SAR408701	CEACAM5	NCT05245071	Recruiting	II	38	ORR	Jun 2022
SAR408701	CEACAM5	NCT05703555	Recruiting	II	60	AEs	Feb 2023
SAR408701	CEACAM5	NCT02187848	Not recruiting	III	263	AEs	Nov 2020
XMT-1536	NaPi2b	NCT03319628	Recruiting	Ib/II	444	ORR	Dec 2017
XMT-1536	NaPi2b	NCT04396340	Not recruiting	I/II	120	DLT	May 2020
MRG003	EGFR	NCT04838548	NA	II	90	PFS; OS	Sep 2020
ABBV-221	EGFR	NCT02365662	Terminated	I	46	AEs	Jan 2015
MGC018	B7-H3	NCT03729596	Terminated	I/II	143	SAEs	Mar 2023
CX-2009	CD166	NCT03149549	Recruiting	I/II	99	ORR	Jun 2017
Cofetuzumab Pelidotin	PKT7	NCT04189614	Not recruiting	I	60	ORR	Feb 2020
SC-002	SCLC	NCT02500914	Terminated	I	35	MTD	Aug 2018
IMGN901	CD56	NCT01237678	Terminated	I/II	181	PFS; MTD	May 2015
IMGN901	CD56	NCT00346385	Completed (NA)	I	97	AEs	Oct 2011
Glembatumumab vedotin	GPNMB	NCT02713828	Terminated	I/II	13	DOP, PFS, OS	Apr 2016
BAY94-9343	MSLN	NCT03455556	Terminated	I	49	MTD	Aug 2018
BAY94-9343	MSLN	NCT02839681	Terminated	II	55	ORR, PFS, OS	Jul 2016
BMS-986148	MSLN	NCT02341625	Terminated	I/II	126	AEs	Jun 2015
RG7841	LY6E	NCT02092792	Completed (NA)	I	42	DLT	Apr 2014
PF-06263507	TPBG	NCT01891669	Terminated	I	26	DLT	Aug 2013
BL-B01D1	EGFR × HER3	NCT05194982	Recruiting	I	96	DLT, MTD	Nov 2021
BL-B01D1	EGFR × HER3	NCT05924841	Not yet recruiting	II	100	PFS, DCR, DOR	Jul 2023
BL-B01D1	EGFR × HER3	NCT05880706	Not yet recruiting	II	42	ORR	Jul 2023
BL-B01D1	EGFR × HER3	NCT05393427	Recruiting	I	26	DLT, MTD	Feb 2022
BL-B01D1	EGFR × HER3	NCT05470348	Recruiting	I	36	DLT, MTD	Aug 2022
BL-B01D1	EGFR × HER3	NCT05803018	Recruiting	I/II	32	ORR	Apr 2023
BL-B01D1	EGFR × HER3	NCT05785039	Recruiting	II	32	ORR	Apr 2023
SYSA1801	Claudin 18.2	NCT05009966	Recruiting	I	272	DLT	Sep 2021
TORL-1–23	Claudin 6	NCT05103683	Recruiting	I	90	MTD	Nov 2021
CBP-1008	TRPV6/FRα	NCT04740398	Recruiting	I	143	AEs, MTD	Mar 2019

*DLT* dose-limiting toxicity, *MTD* maximum tolerated dose, *AE* adverse event, *Cmax* maximum plasma concentration, *TEAE* treatment-emergent adverse event. *NA* not available

**Table 7:** Constituents of ADCs used in lung cancer treatment

ADC drug name	Payload	Linker	Antibody
T-DM1	Emtansine	Thioether linker	Trastuzumab
T-DXd	Deruxtecan	DXd linker	Trastuzumab
ADCT-402	Pyrrolobenzodiazepine	Di-thiomaleimides	Loncastuximab tesirine
HER3-DXd	Deruxtecan	Tetrapeptide linker	Patritumab
MRG003	MMAE	Val–Cit	MMAE
Teliso-V	MMAE	Mc-vc-PAB	ABT-700
DS-1062a	Topoisomerase I inhibitor	Tetrapeptide linker	Datopotamab
IMMU-132	Topoisomerase I inhibitor	CL2A linker	Sacituzumab
ABBV-399	MMAE	Valine glutamic acid linker	ABT-700
ABBV-181	PBD dimer	Val-Ala linker	DLL3 antibody
Rova-T	PBD	Mc-vc-PAB linker	SC16
BA3011	MMAE	Val–Cit	Sggc-Fc
TIVDAK	MMAE	Enzyme-sensitive linker	TF antibody
PF-06647020	Auristatin-0101	Val–Cit	Cofetuzumab
SAR-408701	DM4	SPDB	SAR408377
XMT-1536	MMAF	Succinimidyl 4-(N-maleimidomethyl) Cyclohexane-1-carboxylate	NaPi2b antibody
XMT-1592	MMAF	Dolasynthen	NaPi2b antibody
MRG003	MMAE	Val–Cit	EGFR antibody
MGC018	Docamycin	Val–Cit	Omburtamab
CX-2009	DM4	Enzyme-sensitive linker	Praluzatamab
IMGN901	Maytansine DM1	Cleavable SPP linker	CD30 antibody
XMT-1522	MMAF	Fleximer polymer linker	HT-19
SG	SN-38	Noncleavable linker	Sacituzumab
Glembatumumab vedotin	MMAE	Mc-vc-PAB	Glembatumumab
Anetumab ravtansine	DM4	Cyclohexane-1-carboxylate	MF-T
Tisotumab vedotin	MMAE	Enzyme-sensitive linker	TF-011
EnaV	MMAE	Mc-vc-PAB	AXL-107
BL-B01D1	ED04	Enzyme-sensitive linker	EGFR and HER3 antibody
SYSA1801	LND002	pH-sensitive linker	CLDN18.2 antibody
TORL-1–23	MMAE	Val–Cit	CLDN6 antibody
CBP-1008	MMAE	MC-VC	FRα and TRPV6 antibody
ABBV-221	MMAE	Val–Cit	EGFR antibody
PF-06263507	MMAE	MC linker	TPBG antibody
BMS-986148	Duocarmycin	Val–Cit	MSLN antibody

*MMAE* monomethyl auristatin E, *MMAF* monomethyl auristatin F, *PBD* pyrrolobenzodiazepines

## Peptide–drug conjugates (PDCs)

Despite three generations of ADC development, many unresolved issues remain. The first-generation ADCs contained mouse-derived antibodies and uncleavable linkers. Their disadvantages included insufficient cytotoxicity and low expression of loci. The drawbacks of the second-generation ADCs included DARs that were too low or too high, narrow treatment windows and low effectiveness. The disadvantages of third-generation ADCs include the difficulty of replicating conjugation technology and the insensitivity of cancers to microtubule protein inhibitors [287]. PDCs have the advantages of easy synthesis and purification and low production costs, and they are the most promising type of drug conjugate for achieving therapeutic breakthroughs after ADC [288, 289].

Peptides, as ligand analogs, are characterized by strong targeting ability and the ability to assemble with other drugs. Assembling peptide analogs with chemotherapeutic drugs can produce PDCs with targeted delivery effects [160, 290]. Compared with ADCs, PDCs have the advantage that peptides are easier to synthesize and purify than antibodies are, leading to lower production and transportation costs. Peptide structural modification can facilitate drug design to increase bioavailability, binding affinity, and stability. Additionally, peptides have lower molecular weights than antibodies and thus can more easily penetrate the tumor matrix and enter tumor cells. The structure and composition of PDCs are simpler, and the immunogenicity is lower, which corresponds to a lower probability of an immune stress response in the body. Additionally, PDCs can be eliminated by the kidneys, which results in lower liver toxicity and higher safety. The main indications for PDCs include esophageal tumors, brain tumors, lung cancer, gastric tumors, ovarian tumors, multiple myeloma, pancreatic tumors, and advanced solid tumors [291], making PDCs a promising new generation of targeted anticancer drugs after small-molecule drugs, mAbs and ADCs.

In recent years, the U.S. FDA has approved clinical trials of several tumor-targeting peptide compounds as potential drugs [292]. The selection of tumor protein targets is a major focus of research on tumor-targeting peptides and is directly related to whether a given peptide can be used as an antitumor drug. With the rapid development of X-ray crystallography technology, computer systems, and component technology, great progress has been made in computer-aided drug design [293–297]. Tumor-targeting PDCs have become a new research focus for the development of antitumor drugs in recent years, as they can overcome the disadvantages of conventional chemotherapeutic drugs, particularly by providing increased selectivity between normal and tumor cells [298]. According to statistics from the U.S. clinical trial database, Aeterna Zentaris has conducted 5 phase II/III clinical trials for AEZS-108, which targets breast cancer, endometrial cancer, prostate cancer, and urothelial carcinoma; MolMed has conducted 12 phase II/III clinical trials for NGR–hTNF, which targets colon cancer, ovarian cancer, NSCLC, small-cell lung cancer, malignant thymic epithelial tumors, and metastatic adult soft tissue sarcomas [299]. However, tumor-targeting PDCs have common drawbacks, such as rapid in vivo metabolism and weak drug stability. Rational drug design using appropriate targeting peptides, linker molecules, and cytotoxic payloads can mitigate these problems to some extent [300].

### Tumor-targeting peptides

Tumor-targeting peptides are predominantly synthesized via solid-phase peptide synthesis. The payload, which is the active pharmaceutical ingredient, is manufactured through processes such as synthesis, extraction, or fermentation. The linker is designed with a minimum of two functional groups to facilitate the covalent connection of the tumor-targeting peptide and the payload through chemical synthesis. Tumor-targeting peptides can specifically recognize tumor blood vessels or tumor-related receptors to achieve targeting. With advancing research techniques, many tumor-targeting peptides have been discovered [124, 126, 301–304]. The accumulation of PDCs in tumors and normal organs relies primarily on tumor-targeting peptides, which play a crucial role in molecular targeting. Compared to alternative drug delivery systems, the elimination of extraneous components from molecular drug delivery systems increases the clinical efficacy and safety in cancer patients, thereby maximizing the therapeutic outcome [305, 306].

#### PDCs targeting CD13

Mammalian aminopeptidase N (APN)-CD13 is an ectoenzyme found on the surface of cells and is overexpressed in lung cancer [307]. The peptide Asn-Gly-Arg (NGR) is a tumor-targeting peptide that is upregulated during angiogenesis and the formation of new blood vessels. It specifically binds to vascular cells that express APN. Currently, there are two fusion protein drugs based on the concept of PDCs that incorporate the NGR-targeting peptide: NGR–human tumor necrosis factor (hTNF) and truncated tissue factor (tTF)–NGR [308]. These drugs are currently undergoing clinical studies. In tTF–NGR, the active payload is the external domain of tTF, while Gly-Asn-Gly-Arg-Ala-His-Ala serves as the tumor-targeting peptide connected to the C-terminus of tTF. tTF–NGR has shown acceptable tolerability in low-dose clinical applications and has successfully reduced tumor perfusion. Phase I clinical trials of this treatment for solid tumors, including lung cancer, are currently underway. Although fusion protein drugs do not exactly imitate PDCs since the payload is directly linked to the tumor-targeting peptide, both NGR–hTNF and tTF–NGR exhibit targeting and therapeutic characteristics identical to those of PDCs.

#### Integrins

Integrins regulate various steps in tumor cell migration and invasion and affect tumor cell growth and survival during tumor cell escape and blood/lymphatic vessel infiltration [309]. Integrins consist of 24 heterodimeric cell adhesion receptors, each consisting of α and β subunits. The extracellular region of the α chain includes four extracellular domains. Arg-Gly-Asp (RGD) can bind to a total of 8 integrins [310]. Among them, ανβ3, ανβ5, α5β1 and ανβ6 are associated with cancer progression and metastasis. RGD has the highest affinity for ανβ3 and ανβ5, neither of which is expressed in normal tissues. Therefore, targeting integrins with RGD-based ligands is highly important for specifically targeting tumor cells that overexpress integrins in antiangiogenic therapy.

Several PDC candidates targeting RGD peptides, including [^18^F]Fluciclatide, [^18^F]RGD-K5 [311], and ^68^Ga-NOTA-bombesin (BBN)–RGD, have recently entered clinical trials as positron emission tomography (PET) tracers. Although RGD peptide sequences have many advantages, they also have several shortcomings. RGD-based anticancer drugs and imaging agents can target and bind to integrins to inhibit tumor angiogenesis, but they can also promote tumor cell adhesion, spreading and migration [312–314].

#### PDCs targeting SST

There are five subtypes of SST receptors, which are widely distributed in the brain, pancreas, and pituitary tissues. Natural SST is rapidly degraded by enzymes, and the half-life (t½) is short, less than 3 min after intravenous injection. To date, various SST analogs, such as octreotide (t½ = 2 h), have been developed as prodrugs. The affinity of octreotide for the SST2, SST5, SST3, SST1, and SST4 receptors is high (IC_50_ 0.38–0.60 nmol/l), relatively high (IC_50_ 6.3–7.0 nmol/l), moderate (IC_50_ 7.1–34.5 nmol/l), low (IC_50_ 280–1140 nmol/l), and > 1000 nmol/l, respectively. The use of octreotide and its analogs as tumor-targeting peptides and radioactive isotopes or cytotoxic molecules as effective payloads can achieve the therapeutic/diagnostic purpose of targeting SST2 receptors on tumor cell surfaces. Currently, several PDCs based on octreotide as a tumor-targeting peptide, including diagnostic agents and imaging agents such as ^111^In-DTPA-octreotide, ^99m^Tc-HYNIC/EDDA-^3^Tyr-octreotide, ^68^Ga-DOTATOC, ^68^Ga-DOTATATE and ^177^Lu-DOTATATE, are on the market [315–317].

### Other receptors

Lung cancer is closely associated with the overexpression of membrane type-1 matrix metalloproteinase (MT1-MMP). Consequently, MT1-MMP is considered a potential prognostic biomarker of lung cancer and is linked to unfavorable prognosis. Compared to ADCs, BT1718, an MT1-MMP-targeted PDC, has a low molecular weight and a favorable distribution. As a result, BT1718 can rapidly infiltrate and eliminate tumor cells, achieving a positive therapeutic effect on advanced solid tumors.

Prostate-specific membrane antigen (PSMA) is highly overexpressed in the neovasculature of prostate tumor cells and most solid tumors but not in normal blood vessels. After intravenous administration, PSMA-targeted G202, a soluble thapsigargin prodrug, is metabolized into the active cytotoxic analog of thapsigargin, known as 12-ADT-β-Asp. This mechanism obstructs the nutrient supply to tumor cells, resulting in a high concentration of 12-ADT-β-Asp at the tumor site without causing systemic toxicity.

Hepatocyte receptor A2 (EphA2) expression is generally low in healthy adult tissues but abnormally high in various solid tumors and is associated with poor prognosis. BT5528, a PDC that targets EphA2, can accumulate in tumor tissues at a minimal plasma concentration, increasing the selectivity to eradicate tumor cells while minimizing systemic toxicity.

PDCs have attracted widespread attention due to their ability to significantly improve targeting and ameliorate toxicity and resistance, but many challenges remain. First, the molecular delivery system is administered mainly by injection to prevent degradation in the gastrointestinal tract, but the inconvenience of injections results in poor patient compliance. Second, the in vivo distribution and targeting time of PDCs are limited. The short half-life of tumor-targeting peptides results in a short window of time for effective payload entry into tumor cells. The existing strategies to address this challenge include head-to-tail cyclization, disulfide bond cyclization, substitution of nonnatural amino acids, peptidomimetics, stapled peptides, and bicyclic peptides. However, these strategies must not compromise the binding of tumor-targeting peptides to receptors.

Furthermore, PDCs rely on conditions such as pH, redox status, and enzyme activity in vivo to release the payload. This dependence prevents some payloads from being released as prodrugs or, in some cases, from being released at all. Additionally, payloads modified with functional groups can exhibit significantly reduced biological activity compared to that of prodrugs. Therefore, it is necessary to demonstrate that the active targeting advantage of PDCs can counterbalance the reduced biological activity of the payload. Some candidate drugs have been terminated due to unsatisfactory clinical results, indicating the need to improve the molecular delivery systems.

**Fig. 5:**
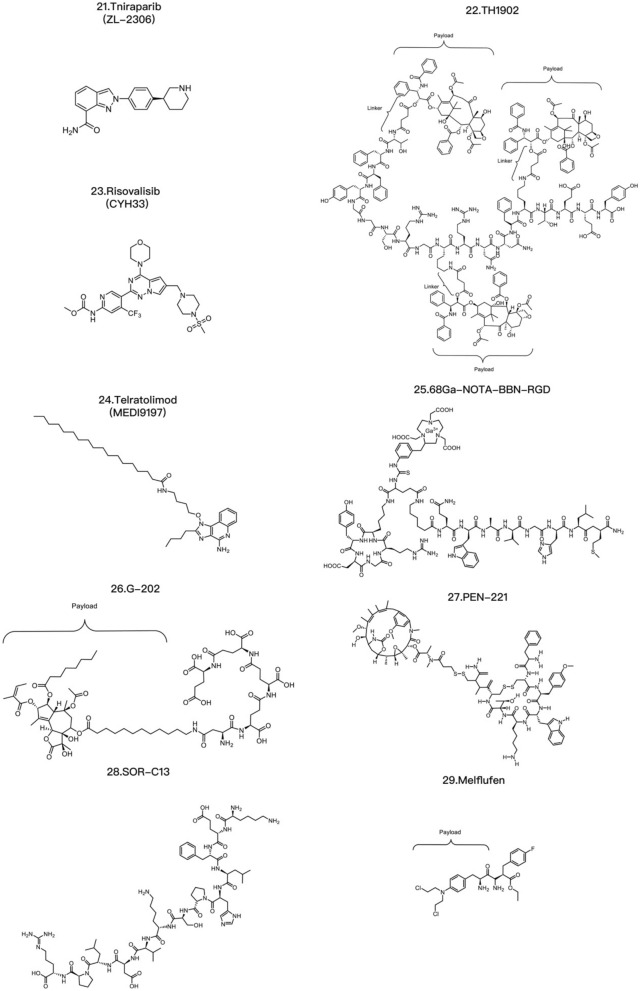

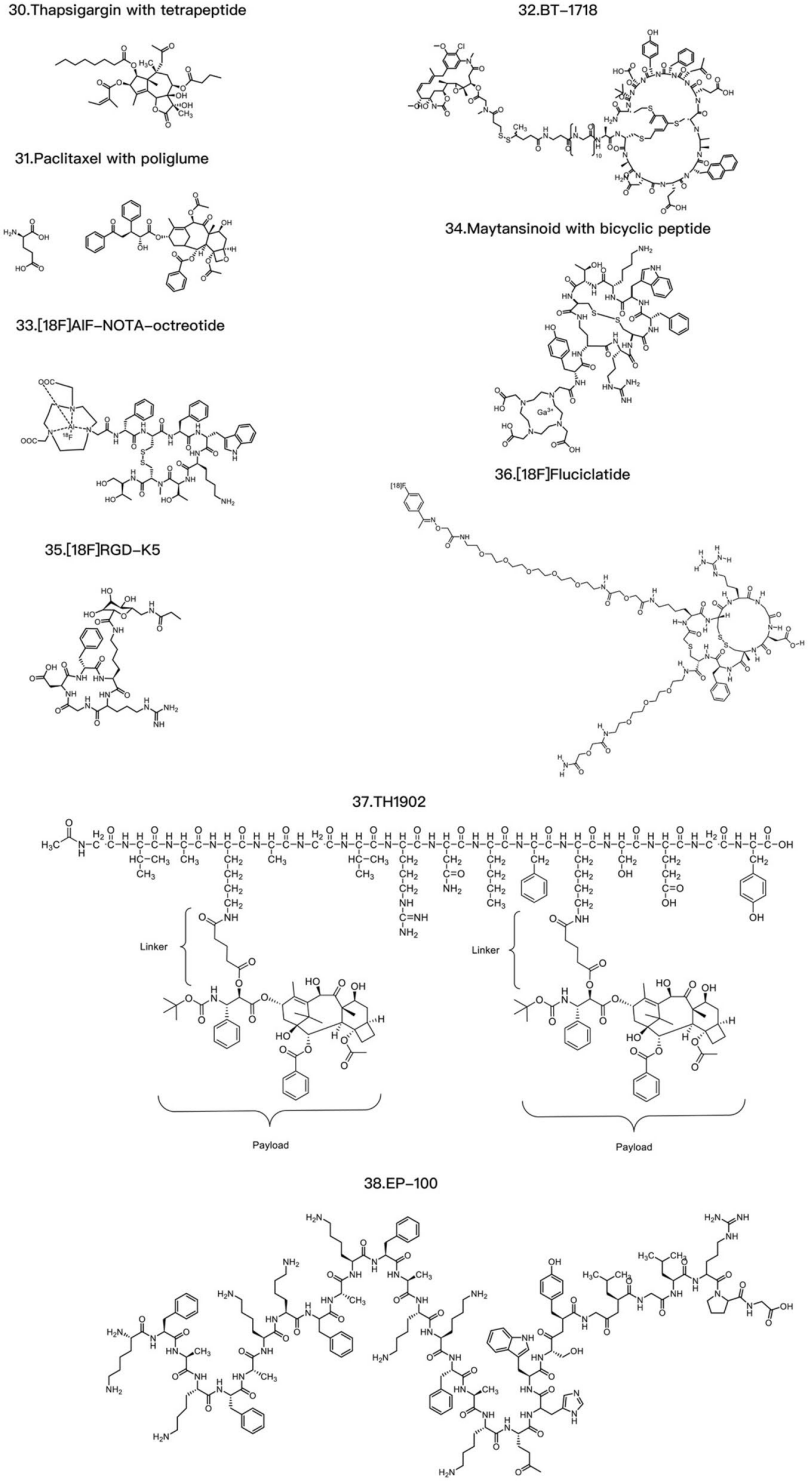
Chemical structures of representative peptide‒drug conjugates (PDCs) in clinical trials for lung cancer treatment

From clinical diagnosis to cancer treatment, peptide-based drug delivery systems are flourishing. Formulation is still considered the key to the drug delivery process, and PDCs can currently satisfy all functional requirements for drug formulations, including absorption, distribution, metabolism and excretion. Compared to nontargeted anticancer drugs applied in clinical practice, molecular delivery systems based on PDCs exhibit significant advantages: prolonged circulation time, increased maximum tolerable doses, elevated drug accumulation in tumor cells, and increased anticancer biological activity. All of the PDCs currently in development are in specific clinical trials for lung cancer [286] (Table 8). The composition of each PDC is presented in Table 9, and the chemical structure of each PDC is shown in Fig. 5.

**Table 8:** Clinical trials of PDC drugs for lung cancer treatment

PDC drug name	Target	NCT number	Status	Study phase	Actual enrollment	Primary endpoint	Start date
ZL-2306	PARP	NCT03516084	Terminated	III	185	PFS	Aug 2018
CYH33	STAT3	NCT04586335	Recruiting	I	350	ORR	Sep 2020
CYH33	STAT3	NCT03544905	Recruiting	I	100	MTD	Jul 2018
MEDI9197	IL-17RA	NCT02556463	Terminated	I	53	MTD	Oct 2018
BT-1718	STn	NCT03486730	Not recruiting	I/II	72	MTD	Jan 2018
GRN1005	LPR1	NCT01679743	Withdrawn	II	20	Not provided	Aug 2012
GRN1005	LPR1	NCT01497665	Terminated	II	16	ORR	Jan 2013
GRN1005	LPR1	NCT00539383	Completed (NA)	I	56	MTD	Mar 2010
BT-5528	CD13	NCT04180371	Recruiting	I/II	288	MTD	Nov 2019
G-202	CD13	NCT01056029	Completed (NA)	I	30	MTD	Dec 2012
PEN-221	CD13	NCT02936323	Completed (Positive)	I/II	89	MTD	Dec 2016
tTF–NGR	CD13	NCT02902237	Completed (Positive)	I	24	MTD	Mar 2017
TH1902	SORT1	NCT04706962	Not recruiting	I	70	MTD	Mar 2021
CBP-1008	Frα and TRPV6	NCT04740398	Recruiting	I	143	AEs	Mar 2019
CBP-1018	PSMA and FRα	NCT04928612	Recruiting	I	170	AEs	Nov 2021
SOR-C13	TRPV6	NCT01578564	Completed (NA)	I	23	Plasma levels of SOR-C13	Jul 2015
Paclitaxel with Poliglumex	PCSK9	NCT00487669	Completed (Positive)	II	14	ORR	Nov 2009
Paclitaxel with Poliglumex	PCSK9	NCT00551733	Terminated	III	450	OS	Dec 2007
Paclitaxel with Poliglumex	PCSK9	NCT00352690	Terminated	II	10	OS	Jun 2008
Paclitaxel with Poliglumex	PCSK9	NCT00269828	Terminated	III	600	OS	Dec 2005
EP-100	GnRH	NCT00949559	Completed (NA)	I	38	Not provided	Mar 2012
Lutathera	–	NCT03325816	Completed (Positive)	I/II	9	MTD	Nov 2017
^[18F]^Fluciclatide	αvβ5 and αvβ3	NCT02193672	Withdrawn	I	0	Not provided	Aug 2014
^[18F]^Fluciclatide	αvβ5 and αvβ3	NCT01176500	Withdrawn	I/II	0	Safety	Nov 2011
^[18F]^RGD-K5	–	NCT00988936	Completed (NA)	II	35	Usefulness	Mar 2012
^[18F]^RGD-K5	–	NCT00743353	Completed (NA)	I	16	Not provided	Jan 2009

*DLT* dose-limiting toxicity, *MTD* maximum tolerated dose, *AE* adverse event, *Cmax* maximum plasma concentration, *TEAE* treatment-emergent adverse event, *NA* not available

**Table 9:** Constituents of PDC drugs

PDC drug name	Payload	Linker	Peptide
ANG1005	Paclitaxel	Succinic acid	Angiopep-2
CBP-1008	MMAE	Amide	CB-20BK
CBP-1018	MMAE	Amide	LDC10B
BT-1718	DM1	Disulfide	MT1-MMP binder
BT-5528	MMAE	Amide	Nectin-4 binder
G-202	Thapsigargin	Amide	DγEγEγEγE
PEN-221	DM-1	Disulfide	fCYwKTCC (2,7 SS)
tTF–NGR	tTF	Amide	GNGRAHA
TH1904	Doxorubicin	Succinic acid	TH19P01
TH1902	Doxorubicin	Succinic acid	TH19P01
SOR-C13	MMAE	Amide	Folic acid
Melflufen	Alkylating agents	Enzymatically cleaved linker	–
Paclitaxel with Poliglumex	Paclitaxel	Ester	Poliglumex
Thapsigargin with Tetrapeptide	Thapsigargin	Ester	Tetrapeptide
Maytansinoid with Bicyclic peptide	Maytansinoid	Disulfide	Bicyclic peptide
Doxorubicin-Tetrapeptide	Doxorubicin	Amide	Tetrapeptide
EP-100	CLIP71	Amino bond	LHRH
^[18F]^AlF-NOTA-octreotide	18F	NOTA	Octreotide
^[18F]^Fluciclatide	18F	PEG	RGD
^[18F]^RGD-K5	18F	NOTA	Cyclo(RGDfK)
68 Ga-NODAGA-E[cyclo(RGDyK)]_2_	68 Ga	NODAGA	E[cyclo(RGDyK)]_2_
68 Ga-NOTA-BBN-RGD	68 Ga	NOTA	Cyclo(RGDyK)和BBN
TH1902	Docetaxel	Succinic acid	TH19P02

*MMAE* monomethyl auristatin E

## Other drug conjugates

### Radionuclide drug conjugates (RDCs)

Radionuclide drug conjugates (RDCs) developed because ADC contain chemotherapy drugs, which may cause a series of toxic reactions. RDCs replace cytotoxic drugs with nucleotides, which can be conjugated with antibodies to form radionuclide–antibody conjugates (RACs) [318]. RDCs are emerging precision tumor therapy drugs that utilize tumor antigen-specific molecular carriers for delivery, accurately targeting radionuclides to tumors for brachytherapy [319]. The mechanisms of the therapeutic effect of RDCs on tumors are as follows: (1) After the radiolabeled antibody specifically targets the membrane antigen on the tumor surface, the radionuclide directly damages DNA, mitochondrial DNA, the cell membrane, etc. The surrounding cells are also exposed to radiation through cross effects. Cell damage leads to the secretion and release of cytokines, ions, ROS, RNS, or exosomes into the extracellular microenvironment. (2) The cytokines and other effector molecules released into the microenvironment bind to cell death receptors, inducing adjacent cancer cell death by a bystander effect. (3) The irradiated cells secrete DAMPs that can bind to the T-cell receptor of antigen-presenting cells, activate the immune system by binding to CD4 or CD8 T cells, and attack remote tumor cells in another type of bystander effect. Radionuclides generally include the β-emitting radionuclides ^131^I, ^90^Y, ^177^Lu, and ^188^Re and the α-emitting radionuclides ^213^Bi and ^211^At. The targets included 4 proteins related to hematological tumors, namely, CD20, CD22, CD33 and CD66, as well as 12 proteins related to solid tumors. Relevant clinical trials are currently being conducted. In recent years, the focus of RDC development has gradually shifted toward the treatment of solid tumors. However, due to issues such as difficulty in delivery caused by abnormal tumor blood vessels and nontarget organ toxicity, the development of RDCs for solid tumors is challenging. The selection of appropriate radionuclides and carriers according to the tumor type and tumor antigen is crucial for optimizing and balancing the therapeutic effect, increasing the dose absorbed by tumors and reducing the toxicity to nontarget tissues. In the past 10 years, multiple clinical trials of RDCs have been published, with 67% for the treatment of nonsolid tumors and 33% for the treatment of solid tumors. Lymphoma accounts for the vast majority (92.5%) of nonsolid tumors in clinical trials of RDCs. Among solid tumors, the types and targets covered by RDCs are more diverse. RDCs in trials for lung cancer treatment include ANG1005, ITM-41, ^111^In-DTPA-octreotide, ^99^mTc-EDDA, ^68^Ga-DOTATATE, ^68^Ga-DOTATOC, Lu^177^ dotatate, ^68^Ga-PSMA-11 and Cu-64 DOTATATE.

### Small-molecule–drug conjugates (SMDCs)

SMDCs are usually composed of targeted molecules, linkers and effector molecules [320]. In fact, due to the excessive segmentation in the field of drug conjugates, there is also crossover between different drug concepts, and PDCs are examples of SMDCs. The largest difference between SMDCs and ADCs lies in the targeting ligand. The ligand of an ADC is a macromolecular antibody that binds to antigens, while the ligand of an SMDC is a relatively low-molecular-weight organic functional group that binds to transporters with high selectivity. Small-molecule ligands also determine the pharmacokinetic characteristics of SMDCs, as they can easily penetrate and spread evenly into tumor tissue without aggregating in either tumors or normal cells. The small amount of off-target drugs will be quickly expelled from the body, which also decreases toxicity to normal cells. The mechanisms of action of SMDCs and ADCs also highly similar. For example, SMDCs targeting folate receptors cannot enter cells through the reduced folate carrier channels by which normal cells absorb folate. Instead, similar to ADCs, they bind to high-affinity folate receptors and enter cells by endocytosis; they are then cleaved and release cytotoxic molecules, exerting a killing effect, while the folate receptors cycle back to the cell surface [321].

PEN-866 is an SMDC developed by Saisheng Pharmaceutical that is currently undergoing phase II clinical trials for the treatment of solid tumors in the United States. PEN-866 carries SN-38, an active metabolite of the topoisomerase inhibitor irinotecan, into the tumor and accumulates in the tumor by the selective binding of the small molecule with the intracellular target heat shock protein 90 (HSP90). SN-38 is cleaved and released over time, preventing adverse reactions caused by systemic irinotecan exposure. Clinical data show that at the recommended dose (175 mg/m^2^), no DLT was observed in patients who received PEN-866, and only one patient experienced uncomplicated G3-grade transient neutropenia. Comparing these results to the occurrence of ≥ grade 3 neutropenia events in 53.8% of patients treated with irinotecan shows that the safety advantage of PEN-866 was significant. Currently, there are several SMDCs in clinical trials for lung cancer treatment, including PEN-866, EC-1456, MBC-11, CBP-1008, vintafolide, BMS-753493, and EC0489EC0225.

### Virus-like drug conjugates (VDCs)

In VDCs, viral capsids designed to form noninfectious protein nanoparticles (VLPs) act as efficient delivery carriers [322]. In some studies, VLPs from human papillomavirus or HPV were selectively attached to the surface of modified heparan sulfate proteoglycans (HSPGs) to target solid tumor cells or metastatic foci instead of normal tissues. AU-001 is a VDC produced by this mechanism, in which virus-like components selectively bind to HSPG. Conjugated infrared light-activated cytotoxic drugs selectively destroy tumor cells under irradiation, leading to acute necrosis of tumor cells and activation of the immune system to produce antitumor responses.

### Antibody–oligonucleotide conjugates (AOCs)

In AOCs, antibodies are used to deliver therapeutic oligonucleotides (siRNAs, PMOs, etc.) to specific cells or tissues, thereby reducing the amount of the drugs needed to treat diseases and addressing the challenges of targeting and oligonucleotide delivery [323]. The conjugation of oligonucleotides with targeted ligands can also improve the pharmacokinetic properties of oligonucleotides and expand their application scope. Technically speaking, AOCs use antibodies as the delivery medium for small molecules, proteins and other functional molecules. Based on this concept, the AOC product AOC1001 was developed for the treatment of ankylosing myotonic dystrophy type 1 (DM1). AOC1001 consists of three parts: a full-length monoclonal antibody targeting transferrin receptor 1 (TfR1), a linker, and siRNA targeting DMPK mRNA. The indication for AOC1001 is DM1. TfR1 is widely expressed on the cell surface and can transport iron into the cell. Muscle cells require a large amount of iron, which makes TfR1 particularly useful for delivering drugs to muscle cells. The design principle of AOC1001 is to treat DM1 by knocking down the expression of mutated DMPK to release Muscleblind-like (MBNL) and enable it to function normally. MBNL proteins are RNA-binding proteins that were first discovered in Drosophila and play important roles in the development of muscles and eyes, as well as in the pathogenesis of human myotonic dystrophy.

### Antibody‒cell conjugates (ACCs)

ACC technology uses a 5' NHS ester ssDNA linker to conjugate the amino groups of antibodies to cell surface proteins, and two linkers bind to form double-stranded DNA, thus completing the conjugation of immune cells and antibodies [324]. ACCs are similar to CAR-T cells in that they provide targets for cell therapy. The difference is that ACCs require only a chemical reaction for conjugation and do not require genetic modification. This powerful cell therapy approach has the potential to significantly increase the efficacy of NK cells by unlocking multiple receptor signaling pathways, such as γδ T cells, which have the ability to recognize T cells and participate in tumor killing [325]. This approach may enable ACC NK therapy to overcome the challenges of effectively targeting solid tumors with cell therapy. The two ACCs currently under development are ACE1702 and ACE1655.

### Immune-stimulating antibody conjugates (ISACs)

The technical requirements of ISACs are similar to those of ADCs, except that the ISAC payload is a congenital immune agonist or regulator with the ability to transform immunologically cold tumors into immunologically hot tumors [326]. ISACs can activate immune killing and therapeutic sensitivity by modulating immune stimulation and the microenvironment [327]. The drugs used in this approach mainly include the Toll-like receptor agonist (TLR) class ISACs SBT6050, SBT6290 and BDC-1001 [328]; the STING agonist ISAC XMT-2056 [329]; and the Treg cell regulatory ISAC ADCT-301 [330, 331]. The current core candidate drug BDC-1001 is a Boltbody-based drug™. The immune-stimulating antibody of this platform is conjugated with Bolt’s proprietary TLR 7/8 double agonist through a noncleavable linker, which is biologically similar to the anti-HER-2 drug trastuzumab and is used to treat HER2-positive solid tumors. Another SMDC in clinical trials for lung cancer treatment is BDC-2034.

### Antibody fragment–drug conjugates (FDCs)

As the name suggests, FDCs use smaller antibody fragments instead of larger antibody molecules [332]. It is generally believed that antibody fragments are relatively easy to detect and that a higher DAR can be achieved using biotechnology [333]. Compared with ADCs, FDCs have the following advantages: the ability to maximize drug efficacy by promoting higher DARs for the delivery of many active drug molecules; small size, which can enable rapid and uniform tumor penetration and thus faster therapeutic effects; rapid clearance from normal tissues and the circulation because of the small size and lack of Fc [334]; a lack of unnecessary molecular interactions that inhibit drug activity; suitability for most antibody fragment forms, which results in high versatility; the ability to reverse engineer the entire mAb and ADC to facilitate drug conjugate manufacturing, increase solubility, and improve the formulation for the next-generation FDC while retaining the stability and binding function of the scFv; and improved pharmacokinetics/kinetics of the entire mAb. As a next-generation cancer treatment method, FDCs can overcome many limitations of existing treatment options and have great market potential.

### Antibody–degrader conjugates (ADeCs)

ADeCs are currently in the early development stage. The technical principle is the use of protein degradation agents as payloads, combining the tumor specificity of ADCs and the applicability of PROTAC molecular catalysts for the treatment of solid tumors with low target protein expression. A representative ADeC is ORM-5029, developed by Orum Therapeutics. This drug, like ADCs, shares the ability to specifically target tumor cells and can accurately deliver its payload of a new protein-degrading agent to the cell interior to degrade intracellular target proteins. In addition, drugs such as AnDC-0003, AnDC-multiple, TD-0001, and IO-0001 are still under development for the treatment of solid tumors, including lung cancer.

### Aptamer–drug conjugates (ApDCs)

In ApDCs, the antibody in an ADC is replaced with an aptamer. The linker connects the aptamer with the drug molecule, which exerts a therapeutic effect. The aptamer serves as a recognition ligand, guiding the therapeutic drug to a disease site or regulating the biological function of targeted biomarkers [335]. Nucleic acid aptamers are oligonucleotide sequences identified using live cell-based index enriched ligand system evolution technology (Cell SELEX) that can bind to various targets with high affinity and specificity [336]. Compared with antibodies, aptamers have many advantages: (1) the high efficiency of aptamer screening, which takes only a few days to several months; (2) the ability to bind toxins or antigens with low immunogenicity for which corresponding antibodies cannot be found; (3) relatively mature solid-phase synthesis technology, with low cost and small batch differences; (4) ease of modification; (5) better thermal and chemical stability; (6) a smaller molecular weight and thus better tissue permeability; and (7) almost no immunogenicity, with no immune side effects. Aptamers are often used in combination with various therapies, such as chemotherapy, phototherapy, toxins, gene therapy, and vaccines. To date, researchers have designed and developed various nucleic acid ApDCs and nucleic acid aptamer-functionalized nanomedicines and have confirmed their potential to significantly promote drug enrichment in tumor lesions. Despite these unique advantages, the sensitivity of nucleic acid ApDCs to nucleases results in short half-lives in vivo, and nonspecific protein adsorption causes nucleic acid aptamer-functionalized nanodrugs to have poor pharmacokinetic behavior. These problems limit the implementation of antitumor drugs based on nucleic acid aptamers in vivo [337]. One study revealed that tumor-targeted chemotherapy achieved by ApDC nanomicelles can increase the antitumor immune response. Therefore, a multivalent ApDC (ApMDC), an amphiphilic terminal dendritic macromolecule composed of hydrophilic aptamers and hydrophobic single dendrites anchored to four anticancer drugs through acid-sensitive junctions, was designed and synthesized. By co-self-assembly with ApMDC analogs, in which the aptamers are replaced by polyethylene glycol, the surface aptamer density of these nanomicelles can be adjusted to optimize the balance between blood circulation time and tumor-targeting ability. The optimized nanomicelles can promote the immunogenic cell death of tumor cells, thereby significantly increasing the tumor-specific immune response to checkpoint blockade in immune-active tumor-bearing mice. Other drugs are still being developed.

**Fig. 6:**
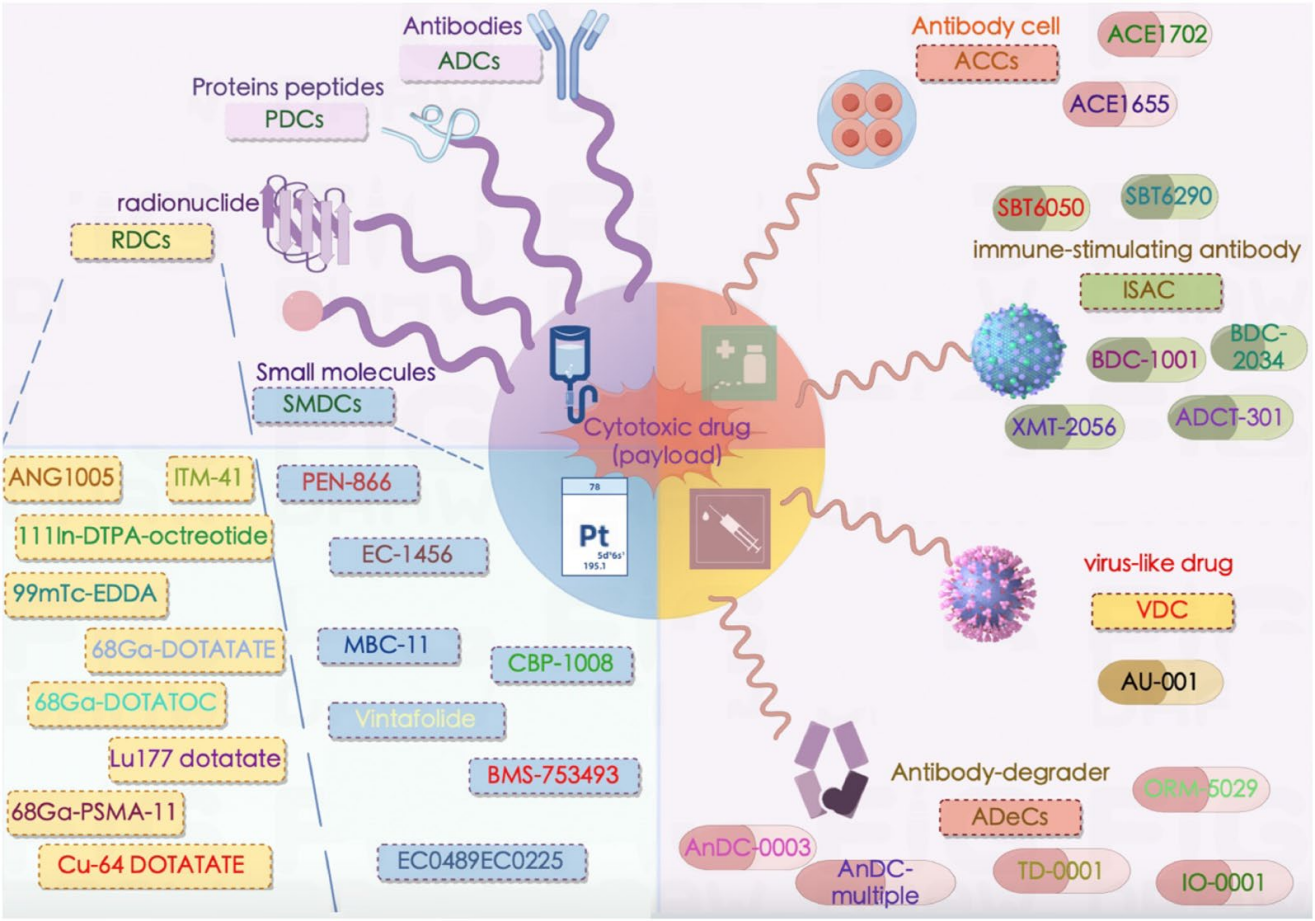
Drug conjugates in trials for lung cancer treatment. *ADCs* antibody‒drug conjugates, *PDCs* peptide‒drug conjugates, *RDCs* radionuclide drug conjugates, *SMDCs* small molecule–drug conjugates, *ACCs* antibody–cell conjugates, *ISACs* immune-stimulating antibody conjugates, *VDCs* virus–like drug conjugates, *ADeCs* antibody–degrader conjugates

**Fig. 7:**
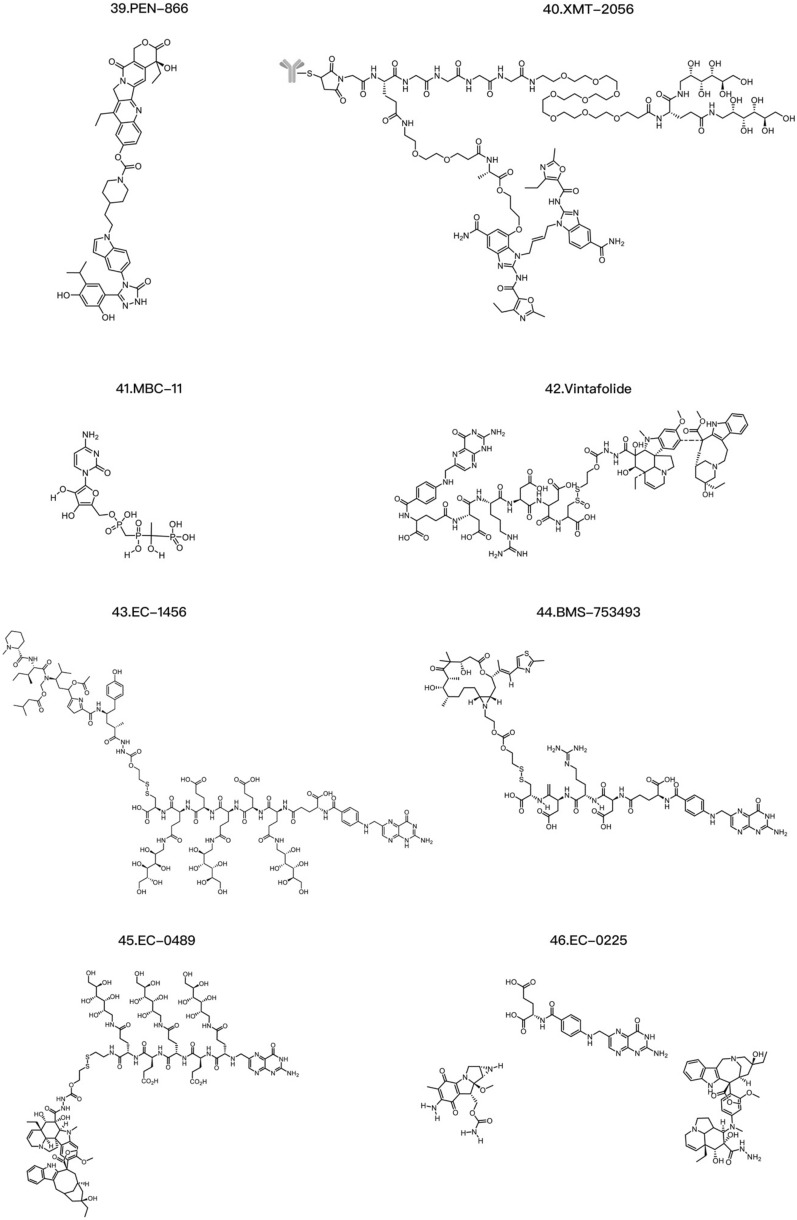
Chemical structures of other representative drug conjugates in clinical trials for lung cancer treatment

Various other drug conjugates currently in clinical trials for lung cancer treatment are presented in Fig. 6. The compositions of these drugs are shown in Table 10, and the chemical structures are shown in Fig. 7.

**Table 10:** Clinical trials of other drug conjugates for lung cancer treatment

Drug name	Target	NCT number	Status	Study phase	Number of subjects	Primary endpoint	Study start date
PEN-866	Hsp90	NCT03221400	Recruiting	I/II	340	DLTs	Aug 2017
EC1456	FR	NCT01999738	Completed (NA)	I	93	Not provided	Oct 2013
MBC-11	Ca +	NCT02673060	Completed (NA)	I	18	AEs	Nov 2015
CBP-1008	TRPV6	NCT04740398	Recruiting	I	143	AEs	Mar 2019
Vintafolide	EGFR	NCT02049281	Terminated	I	3	Cmax	May 2014
Vintafolide	EGFR	NCT01688791	Terminated	I	37	DLTs	Sep 2014
Vintafolide	EGFR	NCT01002924	Completed (NA)	II	1	AEs	Dec 2009
Vintafolide	EGFR	NCT00511485	Completed (NA)	II	43	Clinical benefit	Jul 2009
Vintafolide	EGFR	NCT00308269	Completed (NA)	I	32	MTD	Aug 2007
Vintafolide	EGFR	NCT01577654	Completed (NA)	II	203	PFS	Dec 2013
BMS-753493	–	NCT00546247	Terminated	I/II	26	MTD	Mar 2010
BMS-753493	–	NCT00550017	Terminated	I/II	39	MTD	Dec 2007
EC0489	FR	NCT00852189	Completed (NA)	I	65	MTD	Dec 2011
EC0225	–	NCT00441870	Completed (NA)	I	77	MTD	Feb 2007
ACE1702	HER2	NCT04319757	Recruiting	I	36	AEs	May 2020
SBT6050	HER2	NCT04460456	Not recruiting	I	58	DLTs	Jul 2020
SBT6050	HER2	NCT05091528	Terminated	I/II	2	DLTs	Feb 2022
SBT6290	Nectin4	NCT05234606	Withdrawn	I/II	0	DLTs	Mar 2022
BDC-1001	Toll	NCT04278144	Recruiting	I/II	390	AEs	Feb 2020
XMT-2056	STING	NCT05514717	Suspended	I	171	DLTs	Jan 2021
ADCT-301	Treg cells	NCT03621982	Terminated	I	78	AEs	Nov 2018
ORM-5029	HER2	NCT05511844	Recruiting	I	87	MTD	Oct 2022

*DLT* dose-limiting toxicity, *MTD* maximum tolerated dose, *AE* adverse event, *Cmax* maximum plasma concentration, *PFS* progression free survival

## Toxicities and side effects

The use of drug conjugates in the treatment of lung cancer has been limited by their potential for toxicity [338, 339]. This review summarizes the toxicities of drug conjugates that underwent lung cancer clinical trials in which the primary endpoint events were AEs or serious AEs (SAEs) in Table 11. Although drug conjugates have shown great promise, some studies have resulted in SAEs, and in the NCT03245736 study, 100% of patients experienced other (not including serious) AEs. Nervous system disorders were the most common side effects, and other AEs included blood and lymphatic system disorders; metabolic and nutritional disorders; and respiratory, thoracic and mediastinal disorders. However, the all-cause mortality in this trial was 0. In addition, all of the patients in the NCT02001623 trial experienced AEs, and the most common side effect was nausea. Overall, the all-cause mortality was within acceptable limits. Several lung cancer clinical trials have had primary endpoint events of AEs/SAEs (NCT00346385, NCT02673060, NCT01002924, NCT02277717, NCT02874664, NCT02552121, and NCT03913741); however, the results from these trials have not been disclosed. It is necessary to fully understand the adverse reactions caused by drug conjugates and to establish corresponding safety management strategies and evaluation methods. In addition to tumor therapy, drug conjugates are still in clinical trials and preclinical testing for nontumor indications such as immunity and infection [220].

**Table 11:** Adverse events in clinical trials of drug conjugates for lung cancer treatment

Drug name	Type	NCT number	Time frame	Number of AEs	All-cause mortality	Number of other AEs	Description
Rova-T	ADC	NCT03543358	Up to 13.6 months	Not specified	1 (33%)	1 (33%)	Gastrointestinal disorders; psychiatric disorders
TIVDAK	ADC	NCT03245736	Day 1 to week 24	1 (20%)	0 (0%)	5 (100%)	Nervous system disorders
TIVDAK	ADC	NCT02001623	1 to 249 days	6 (40%)	1 (7%)	15 (100%)	Nausea

*ADC* antibody drug conjugate, *AE* adverse event

Because many of the toxicities associated with drug conjugates are dose related, researchers have expended substantial effort to optimize doses and administration patterns to improve the therapeutic indices of drug conjugates. At present, typical dose optimization strategies include adjustment of the upper limit of dose, adjustment of the upper limit of treatment duration, graded dose administration, patient treatment response-guided dose adjustment, and random-effect dose research [340].

The optimization of drug conjugate structures is important for maximizing the efficacy and safety of formulations and can affect tolerance [341]. In addition to common drug conjugates, probe–drug conjugates can reduce the incidence of targeted and nontumor toxicity [342]. Because the vast majority of payloads are released in the circulatory system, the toxicity of drug conjugates is currently similar to that of chemical drugs. The most important aspects of optimization are the tumor-targeted delivery of drug conjugates and the use of cleaved linkers to increase the bystander effect [343]. Reducing the risk of AEs after drug conjugate treatment is an important step in clinical management. The genomic parameters of drugs may also affect their reactivity. The inclusion of drug genome maps in early drug design trials may be a reasonable approach to ensure that safety is not excessively affected by genetic variations among populations or individuals.

## Outlook

### Challenges in drug conjugate development

The currently approved drug conjugates are much more potent than conventional chemotherapeutic agents. The development and application of drug conjugates could have a unique impact on lung cancer treatment. A legitimate question, therefore, is to what extent drug conjugates can optimize conventional cytotoxic chemotherapy, at least for some indications [344]. There are still some challenges, and the current limitations of drug conjugates include cost, drug resistance, and instability.

#### Drug resistance

The mechanisms of drug conjugate resistance are complicated and can involve [340] antigen-related resistance, endocytosis and migration disorders, lysosomal dysfunction, drug efflux pump activity, mutations in target sites, the cell cycle, the PI3K/AKT signaling pathway and apoptosis dysregulation [345]. However, there are currently no effective treatments to counteract drug conjugate resistance [346, 347]. Combination therapy with other drugs, switching to drug conjugates with different targets, or developing new payload drugs are potential approaches to reversing resistance [182, 348, 349].

#### High heterogeneity

Due to the high heterogeneity and dynamic changes in target antigens expressed by tumor cells, it is necessary to select appropriate drug conjugates targeting tumor tissue-specific antigens. Among the existing ADCs, only a few have shown promising efficacy in target-enriched patients, such as an ORR of 55% for T-DM1 and T-DXd in treating advanced HER2-mutated NSCLC. However, these agents have limited efficacy against NSCLC with other mutation types, such as HER2-overexpressing NSCLC. Currently, the majority of ADCs lack effective biomarkers for predicting efficacy. In the future, relevant studies need to be conducted to explore the selection of patients who will benefit from ADCs. In addition, multiple patient-related factors, including baseline organ function, the presence of comorbidities, and polymorphisms of enzymes involved in ADC metabolism, can influence the pharmacokinetics and pharmacodynamics of these drugs. The analysis of patient heterogeneity may facilitate the development of personalized treatment plans and improve outcomes [350].

#### Instability

In general, the presence of lysine and cysteine residues on antibodies provide reactive sites for conjugation [351, 352]. Early ADCs were typically randomly conjugated via lysine or cysteine residues [353], but this approach can lead to many problems [354]. The stability of these drug conjugates is sometimes insufficient, which can cause premature payload release and off-target toxicity [355–358].

#### DAR limitations

The DAR determines the amount of the payload that can be delivered to the tumor, directly affecting the safety and effectiveness of drug conjugates. Simply put, the effectiveness of drug conjugates is directly linked to the DAR. The DAR can be understood as the amount of ammunition carried by the magic bullet ADC, where the higher the value of the DAR is, the stronger the antitumor efficacy. Although a high DAR represents a large drug-loading capacity, a drug conjugate with a high DAR is also more likely to be recognized by the human immune system as a foreign object and cleared by the body, thus reducing the effectiveness. A high DAR can also easily lead to drug release in the circulatory system, resulting in high toxicity. Therefore, the DAR of most drug conjugates is limited to 2–4.

#### Rapid intracellular disintegration

Rapid disintegration has a critical impact on drug conjugates. The cytotoxic drugs released by the cleavage of a cleavable linker can penetrate the cell membrane and kill the surrounding tumor cells via a process called the bystander effect. In contrast, for a noncleaved linker, even if the antibody is degraded by proteases, amino acid residues remain connected to the linker and the cytotoxic drug. The resulting charged metabolites cannot effectively pass through the cell membrane and therefore usually do not exert a bystander effect.

### How to address these challenges

In the long term, the development of ADCs is mainly aimed at updating linker payloads, but in the short term, antibody forms, including monoclonal antibodies, double antibodies, multiantibodies and existing linker payloads, remain dominant. To address the above challenges, there are currently numerous systematic treatment strategies to ensuring safety while improving treatment efficiency.

#### Overcoming drug resistance

Dual-payload ADCs may be a promising class of drugs to address the clinical challenges of tumor heterogeneity and drug resistance [359]. By accurately controlling the ratio of the two drugs and simultaneously delivering two synergistic toxins to cancer cells, the overall therapeutic effect can be increased, resulting in a higher response rate. Simultaneously, due to the different mechanisms of action of the toxins, the incidence of drug resistance is significantly reduced [360]. For example, in preclinical studies, a single anti-HER2 ADC that includes both MMAE and MMAF [273, 361] exhibited significantly higher antitumor activity than the simultaneous administration of corresponding single-toxin ADCs and even achieved complete remission [362]. However, the design of dual-toxin drug conjugates has not yet been validated in clinical trials. Due to the potential for synergistic (1 + 1 > 2) toxicity, the safety phase is an important focus.

The administration method can also affect the occurrence of drug resistance. In early clinical trials, ADCs were mainly administered as single drugs. Currently, treatment options that combine conventional chemotherapy and other targeted drugs are being explored in clinical practice. In addition, the order of treatment may be an important factor. One study revealed that patients who had previously received trastuzumab/pertuzumab had a worse response to T-DM1 than did those who had not received these two antibodies [363]. In a preclinical T-DM1-resistant model, however, the combination of trastuzumab and pertuzumab was found to be effective [364].

Combinatorial strategies are under investigation to assess the efficacy of drug conjugates delivered in association with partner drugs [365]. Combinations of ADCs with TKIs directed against the same target to increase internalization or overcome TKI resistance or with immunotherapeutic agents for potential synergistic effects in lung cancer can also be evaluated as strategies [366, 367].

#### Solving the heterogeneity issue by target selection and the use of bispecific antibodies (BsAbs)

Strategies for using drug conjugates in lung cancer treatment involve either biomarker-driven or biomarker-agnostic approaches. Different expression levels and cutoff values might affect the efficacy of treatment across different trials. Conversely, HER2 mutations, not HER2 overexpression, have been associated with the response of lung cancer to anti-HER2 ADCs [368], and stronger established drivers might represent better therapeutic targets [369].

According to the latest research reports, combining BsAb technology with ADC technology is currently a new direction in the field of ADCs [370, 371]. At present, there are two main BsAb design strategies: (1) Many targets, such as HER2, have promising tumor expression profiles, but poor internalization and poor lysosome transport limit their full potential as effective ADC targets. An ADC with dual specificity for the two nonoverlapping epitopes of the HER2 protein has been designed and is called a biparental ADC. This structure can increase the cross-linking of cell surface receptors and the aggregation of receptors, thus promoting the internalization and lysosome delivery of ADCs and thereby increasing their efficacy. However, it should be noted that not all nonoverlapping antibodies are equally effective at promoting internalization and lysosome transport, and specific epitopes and spatial directions can even reduce lysosomal transport. In addition, there are some designs that achieve the same function by combining weak internalization targets with strong internalization targets to form bispecific ADCs. (2) To increase the selectivity for tumors over normal tissues, an appropriate combination of two specific ADCs with optimized affinity can be selected, which expanding the therapeutic indices and increases the safety and effectiveness of ADCs. In addition, antigen selection is critical to drug efficacy, and several factors need to be considered in the selection of target antigens: the degree of antigen expression in tumors and healthy tissues; the physiological function of antigens in normal cells and tumor cells; the endocytosis of the antigen and the mechanism involved; if, where and how the antigen is released; the potential impact of antigen shedding on the effectiveness of the ADC; and the antigenic cycle and its influence on the mechanism of ADC action.

#### Increasing stability by adjusting the structure of drug conjugates

Ideally, drug conjugates can maintain their integrity and stability in the blood circulation before entering the target cell, and many methods, including conjugation site selection and linker modification, have been developed to increase drug conjugate stability. In fact, it has been reported that less than 1% of administered ADCs reach human tumors, and the rest may cause unnecessary toxicity. Usually, each component can be modified to increase stability [372, 373], but research has shown that adjusting the conjugation sites and the length and steric hindrance of the linker are more effective general methods [374, 375]. By selecting conjugation or attachment sites with high steric hindrance, spatial shielding by the antibodies can be established [376].

Linkers influence the stability and pharmacokinetics (PK) of a given drug conjugate [377], and linkers can be selected for tumor-specific release, allowing drug release in both the tumor microenvironment and tumor cells without affecting the half-life of the drug conjugate in circulation. Due to the presence of tissue proteases in the tumor microenvironment, peptide linkers that are sensitive to tissue proteases have this characteristic. Improvements in linker development could include the use of (1) payload-masking linkers [378], (2) hydrophilic linkers [379], (3) branched linkers to increase the DAR [380], (4) tandem cleavage linkers, and (5) dual-sensitivity linkers [381]. Payload modifications that might increase the therapeutic benefit of next-generation drug conjugates include the creation of (1) prodrug-based payloads to mitigate off-tumor toxicity [382, 383], (2) hydrophilic cytotoxic payloads [384, 385], and (3) bifunctional payloads to increase antitumor efficacy [386, 387].

#### Raising the DAR by modifying the drug conjugation mode and linker

Concerning DARs and pharmacokinetic characteristics [388, 389], most current drug conjugates use highly potent cytotoxic warheads, which generally produce the expected effect when the DAR is 2–4 [390]. Through modification of the conjugation mode and linker, T-DXd and sacituzumab govite can achieve a DAR of approximately 8, indicating that additional cytotoxic molecules can bind to an antibody without affecting its solubility, aggregation tendency, or pharmacokinetic characteristics [391, 392]. This ability has important implications: compounds with lower potency but different mechanisms of action may serve as effective payloads for drug conjugates. Alternatively, using drug conjugates with the same payload but lower DARs might increase efficacy due to the ability to increase the dosage.

#### Guaranteeing rapid intracellular disintegration by targeting mutant proteins

Recent research has indicated that rapid intracellular disintegration critically affects the cytotoxicity of drug conjugates. Compared to wild-type proteins, mutated proteins typically have greater stability against rapid intracellular disintegration but are more prone to internalization and degradation. Thus, drug conjugates targeting mutated proteins may have significant clinical effects [393]. In addition, designing cleavable linkers to increase intracellular disintegration presents a challenge.

## Prospects

**Fig. 8:**
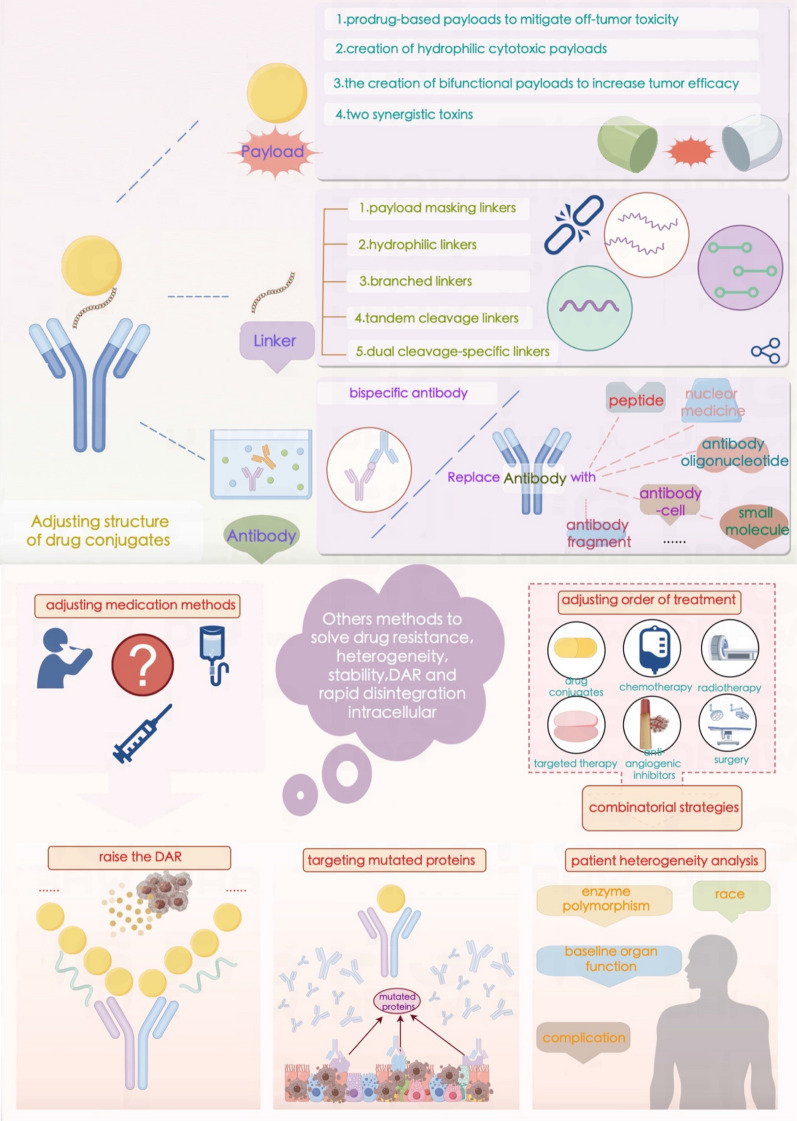
Measures to address the difficulties associated with drug conjugate development and application. *DAR* drug-to-antibody ratio

There are multiple comprehensive treatment methods for lung cancer, and the extent to which drug conjugates offer advantages over traditional treatments is unclear [394]. Although measuring the impact of a novel anticancer agent in the clinic can be difficult, drug conjugates have had a pronounced impact on lung cancer treatment. The approval of 27 drug conjugates by the FDA and the encouraging clinical performance of other drug conjugate candidates have attracted increasing attention in the field, and many studies on lung cancer have shown promising results for drug conjugates. Even if drug conjugate development is more complex than unconjugated drug development [395], the challenges encountered in the development of drug conjugates are being overcome. We expect the number of approved drug conjugates to increase substantially in the coming years, and we anticipate much better treatment effects for lung cancer (Fig. 8).

## Data Availability

Not applicable.

## Abbreviations

ACC: Antibody cell conjugate
ADC: Antibody drug conjugate
ADeC: Antibody degrader conjugate
AOC: Antibody oligonucleotide conjugate
AON: Antisense oligonucleotide
ApDC: Aptamer drug conjugate
DAR: Drug-to-antibody ratio
DCR: Disease control rate
DLL3: Delta-like protein 3
EGFR: Epithelial growth factor receptor
FDA: Food and Drug Administration
FDC: Antibody fragment drug conjugate
GSH: Glutathione
hTNF: Human tumor necrosis factor
ILD: Interstitial lung disease
ISAC: Immune-stimulating antibody conjugate
mAb: Monoclonal antibody
MMAE: Monomethyl auristatin E
MMAF: Monomethyl auristatin F
MTX: Methotrexate
NA: Not available
NSCLC: Non-small cell lung cancer
ORR: Objective response rate
PBD: Pyrrolobenzodiazepines
PDC: Peptide drug conjugate
PET: Positron emission tomography
PFS: Progression free survival.
PSMA: Prostate-specific membrane antigen
RDC: Radionuclide drug conjugate
siRNA: Small interfering RNA
SMDC: Small-molecule drug conjugate
TKI: Tyrosine kinase inhibitor
TTP: Tumor-targeting peptide
VDC: Virus-like drug conjugate
Val–Cit: Valine–citrulline
VEGFR: Vascular endothelial growth factor receptor
WCLC: World Conference on Lung Cancer
